# Functional cooperation between the B-cell receptor and *NOTCH1* in regulating metabolic reprogramming in chronic lymphocytic leukemia

**DOI:** 10.1038/s41375-026-02912-7

**Published:** 2026-03-23

**Authors:** Amelia Fascì, Francesco Edoardo Vallone, Nahal Nabelsi, Elodie Viry, Ilenia Sana, Alessia Morabito, Silvia Seghezzi, Noemi Anna Pesce, Matteo Rovere, Nadia Bertola, Chloé Duculty, Silvia Ravera, Samir Mouhssine, Marta Muzio, Paolo Ghia, Candida Vitale, Marta Coscia, Etienne Moussay, Gianluca Gaidano, John Allan, Richard R. Furman, Jerome Paggetti, Tiziana Vaisitti, Silvia Deaglio

**Affiliations:** 1 Laboratory of Functional Genomics, Department of Medical Sciences, University of Turin, Turin, Italy; 2 Tumor Stroma Interactions, Department of Cancer Research, Luxembourg Institute of Health, Luxembourg, Luxembourg; 3 Division of experimental Oncology, IRCCS Ospedale San Raffaele, Milano, Italy; 4 Medical School, Università Vita-Salute San Raffaele, Milano, Italy; 5 IRCCS Ospedale Policlinico San Martino, Genoa, Italy; 6 Department of Experimental Medicine, University of Genoa and IRCCS Ospedale Policlinico San Martino, Genoa, Italy; 7 Division of Hematology, Department of Translational Medicine, University del Eastern Piedmont and Azienda Ospedaliero-Universitaria Maggiore della Carità, Novara, Italy; 8 Department of Molecular Biotechnology and Health Sciences, University of Turin, Turin, Italy; 9 Department of Hematology, Weill Cornell Medicine, New York, NY USA

**Keywords:** Preclinical research, Leukaemia

## Abstract

Mutations in *NOTCH1*, which occur in ~10% of Chronic Lymphocytic Leukemia (CLL) patients at diagnosis, are typically associated with unmutated (UM) B-cell receptor (BCR) subsets and define patients with earlier treatment need. Using primary CLL cells classified as *NOTCH1* wild-type (CLL/N^WT^) or mutated (CLL/N^M^), both with UM-BCR, we show that BCR stimulation activates the NOTCH1 pathway, upregulating metabolic programs and mitochondrial biogenesis, selectively in CLL/N^M^. These cells display enhanced basal respiration and glycolysis, driven by higher mitochondrial mass, and further increase metabolic activity upon BCR triggering. To directly implicate *NOTCH1* mutations, we engineered an MEC-1 model to generate wild-type (MEC-1/N^WT^) or mutated (MEC-1/N^M^) clones in a UM-BCR background. Here, *NOTCH1* hyperactivation promoted mitochondrial metabolism through TFAM-dependent transcriptional control. Gene expression profiling, metabolic assays, and stable isotope tracing confirmed that MEC-1/N^M^ cells rely on oxidative metabolism, with increased glutamine dependency and strengthened anabolic pathways, leading to augmented proliferation compared to MEC-1/N^WT^. Importantly, CLL/N^M^ cells exhibit a marked vulnerability to glutamine deprivation. Combined inhibition of glutamine utilization and BCL2 triggered rapid apoptosis, providing a rationale for tailored therapeutic strategies in *NOTCH1*-mutated CLL.

**Figure:**
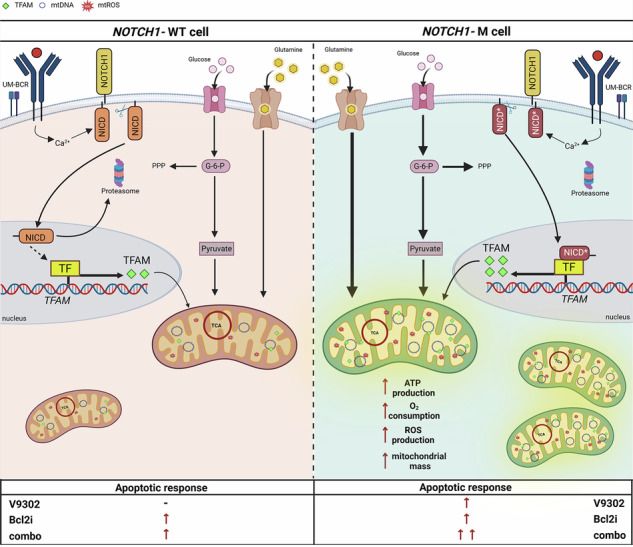
**Representation of the molecular mechanism behind the metabolic reprogramming**. BCR and *NOTCH1* drive a dual metabolic reprogramming of glucose and glutamine pathways. In *NOTCH1*-mutated cells, both glucose and glutamine uptake are positively increased and even more upon BCR stimulation. Glucose is preferentially used to fuel the pentose phosphate pathway, and glutamine the TCA cycle. Concurrently, NICD accumulation, driven by BCR signaling, promotes *TFAM* expression and mitochondrial biogenesis. The resulting increase in mitochondrial mass underpins enhanced ATP production, oxygen consumption, and ROS generation, establishing a glutamine-dependent mitochondrial phenotype. This dependency sensitizes *NOTCH1*-mutated cells to glutamine blockade, which selectively induces apoptosis, further enhanced by combination with BCL-2 inhibition.

**Representation of the molecular mechanism behind the metabolic reprogramming**. BCR and *NOTCH1* drive a dual metabolic reprogramming of glucose and glutamine pathways. In *NOTCH1*-mutated cells, both glucose and glutamine uptake are positively increased and even more upon BCR stimulation. Glucose is preferentially used to fuel the pentose phosphate pathway, and glutamine the TCA cycle. Concurrently, NICD accumulation, driven by BCR signaling, promotes *TFAM* expression and mitochondrial biogenesis. The resulting increase in mitochondrial mass underpins enhanced ATP production, oxygen consumption, and ROS generation, establishing a glutamine-dependent mitochondrial phenotype. This dependency sensitizes *NOTCH1*-mutated cells to glutamine blockade, which selectively induces apoptosis, further enhanced by combination with BCL-2 inhibition.

## Introduction

Chronic lymphocytic leukemia (CLL) is considered an antigen-driven B cell malignancy, with the B cell receptor (BCR) serving as the driving force in disease progression [1]. This is confirmed by the success of Bruton’s tyrosine kinase (Btk) inhibitors in eradicating the disease [2–4]. The variable genes of the heavy chain of the Immunoglobulins (*IGHV*) in CLL cells may be found in germline or mutated configurations, a finding that carries prognostic implications and paves the way for many studies aimed at determining the origin of these cells [5]. Importantly, CLL cells do not bear a common genetic lesion, but are characterized by a complex genetic architecture [6, 7].

The ligand-activated transcription factor *NOTCH1* represents one of the most recurrently mutated genes, with ~10% of patients carrying the mutation at diagnosis [8–11]. It encodes a cell surface molecule that, upon ligand binding, undergoes multiple enzymatic cleavages to generate the NOTCH Intracellular Domain (NICD), which translocates to the nucleus and joins a molecular complex that regulates transcription [12, 13]. In patients with CLL, the vast majority of *NOTCH1* mutations affect the C-terminal PEST domain of the protein, impacting its ability to be ubiquitinated and degraded [14]. This mutated *NOTCH1* is characterized by an extended half-life of the NICD, prolonged NF-κB activity, and sustained transcription of genes involved in cell survival and proliferation [15–18]. Interestingly, the great majority of *NOTCH1*-mutated patients also display unmutated BCR, suggesting a possible relationship between these two pathways [19, 20]. We have previously described a functional interplay between BCR and *NOTCH1* pathways that creates a positive feedback loop amplifying leukemic cell activation [21]. In addition, these two signaling pathways have been recently linked to metabolic reprogramming in CLL. Specifically, CLL cells exhibit enhanced glucose metabolism and oxidative phosphorylation, enabling them to meet increased energy demands [22–24].

*NOTCH1* hyperactivation has been implicated in driving a metabolic shift toward increased glucose uptake and metabolic performances, supporting growth both in CLL and other tumor models [25–27]. In this context, the interplay between aberrant BCR signaling, *NOTCH1* hyperactivation, and metabolic reprogramming represents a critical axis in CLL pathogenesis.

Our findings, obtained in primary CLL cells and verified using a controlled cell line model, demonstrate that *NOTCH1*-mutated CLL cells are characterized by enhanced mitochondrial metabolism, regulated at least in part through the up-regulation of mitochondrial transcription factor A (TFAM). Furthermore, *NOTCH1* mutated primary CLL cells rely heavily on glutamine which is a therapeutic vulnerability.

## Materials and methods

### Reagents and antibodies

Antibodies used in Western Blot (WB) and flow cytometry (FC) are listed in Table S1 and analytical methods in Supplementary Materials and Methods (SMM).

### Patient samples

Patient samples were obtained from CLL peripheral blood. A detailed correspondence between experiments, patient IDs (coded as P#), *NOTCH1* mutational status, statistical comparison of cytogenetic abnormalities and sex distribution is provided in Tables S2A-B. Leukemic cells were purified as described [28].

### Molecular cloning and generation of MEC-1/IgM-UM clones

MEC-1 cells, which carry an *IGHV* 4-59 mutated BCR, were obtained from DSMZ. MEC-1/sIgM^-^ cells, a subclone lacking expression of surface IgM (sIgM) that was constitutively present in the original cells, were sorted by limiting dilution cloning and were reconstituted with the unmutated *IGHV*4-59 sequence of a patient with CLL, as detailed in SMM.

### BCR ligation

For BCR stimulation, the goat anti-human IgM-UNLB (Southern Biotech, Birmingham, AL) was used. Protocols are described in SMM.

### Quantitative real-time PCR (qPCR) and mitochondrial DNA (mtDNA) content determination

RNA extraction, retrotranscription, and analyses were carried out as detailed in SMM. qPCR was used to assess absolute mtDNA levels as an index for mitochondrial mass. *MT-COX4* was used as the standard for mtDNA, and *GAPDH* was used as the nuclear DNA (nDNA) normalizer to calculate the mtDNA/nDNA ratio.

### TFAM down-regulation

For TFAM down-regulation, TFAM, 4392420 – s1400 (Thermo Fisher, Waltham, MA) was used as described in SMM.

### Reactive oxygen species (ROS) production

ROS production was determined using MitoSOX Red (Thermo Fisher), following the manufacturer’s instructions. Fluorescence was measured by BD FACScelesta (Franklin Lakes, NJ).

### MitoTracker staining

Mitochondrial mass was evaluated by FC analysis and by confocal microscopy, as described in SMM.

### Glucose uptake

Glucose uptake was measured using the 2-NBDG Glucose Uptake Assay Kit (Sigma-Aldrich), St. Louis, MO, with fluorescence was measured by BD FACScelesta.

### Seahorse metabolic rate assay

Seahorse assay was performed for measuring Oxygen Consumption Rates (OCR) and Extracellular Acidification Rates (ECAR) using the XFp Mito Stress Test Kit and the XFp Glycolysis (Agilent Technologies, Santa Clara, CA). Full details in SMM.

### Evaluation of glucose consumption, extracellular lactate, ATP synthesis, and OCR

Glucose consumption, extracellular lactate, ATP synthesis, and OCR were measured as described [29, 30]. Details in SMM.

### Stable isotope tracing and metabolite extraction

Stable isotope tracing experiments with [U-^13^C]-glucose and [U-^13^C]-glutamine tracers (Cambridge Isotope Laboratories, CLM-1396) were performed as previously described [31].

### In vitro competition assays

MEC-1/N^WT^ and MEC-1/N^M^ cells were co-cultured at a 1:1 ratio and their density monitored by flow cytometry every 2 days by FC analysis until one of the two populations reached ~80% relative abundance. The relative abundance of the two MEC-1 populations was calculated as a percentage of the morphological gate population and plotted as such. This assay was designed to quantify relative changes in population abundance (competitive fitness) under shared culture conditions and is not intended to infer absolute monoculture proliferation rates.

### RNA sequencing and data analysis

RNA sequencing was performed as described [16]. Details in SMM.

### CUT&Tag

CUT&Tag experiments are detailed in SMM [32].

### Statistical analyses

Statistical analyses were performed with GraphPad 9 (GraphPad Software Inc., La Jolla, CA). All data are plotted as means ± SEM, unless otherwise specified. Statistical significance was tested using unpaired *t* tests, with threshold set at *p* ≤ 0.05.

## Results

### Crosstalk between BCR and *NOTCH1* in primary CLL cells activates genes belonging to metabolic pathways in *NOTCH1*-mutated subset

**Fig. 1:**
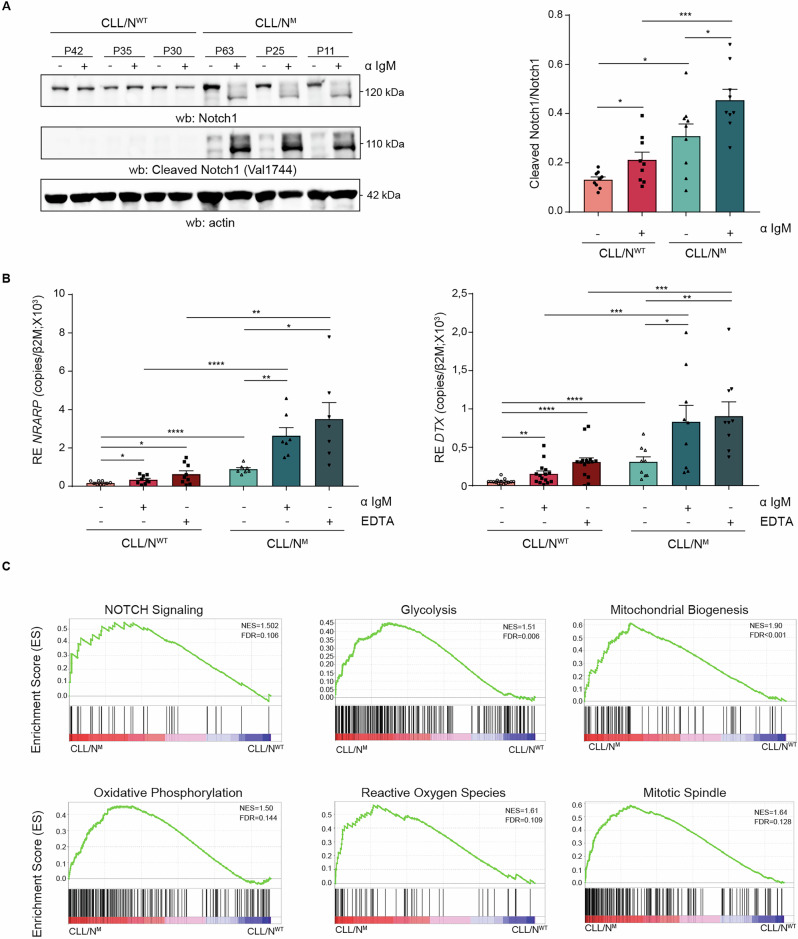
Functional assessment of *NOTCH1* activation and transcriptional signature in CLL/N^M^ cells compared to CLL/N^WT^ cells. **A** Western blot analysis of total and cleaved (Val1744) NOTCH1 in CLL/N^M^ (*n* = 9) and CLL/N^WT^ (*n* = 9) primary cells, either untreated (NT) or stimulated with anti-IgM (aIgM). Actin is shown as a loading control. The right panel shows quantification of cleaved NOTCH1 (Val1744) normalized to total NOTCH1 levels. **B** qtRT-PCR analysis of *NRARP* (left, CLL/N^WT^ *n* = 9 and CLL/N^M^ = 7) and *DTX1* (right, CLL/N^WT^ *n* = 14 and CLL/N^M^ *n* = 9) following stimulation with anti-IgM and/or EDTA, as indicated. mRNA expression was normalized over β-2-microglobulin (*β2M*). **C** Gene Set Enrichment Analysis (GSEA) comparing transcriptional profiles from a down-sampled cohort of CLL/N^M^ (*n* = 50) and CLL/N^WT^ (*n* = 50) patients. Enrichment plots are shown for curated gene sets related to NOTCH1 signaling, glycolysis, mitochondrial biogenesis, oxidative phosphorylation, reactive oxygen species (ROS), and mitotic spindle. Normalized Enrichment Score (NES) and False Discovery Rate (FDR) values are indicated in the top-right corner of each panel. (*p* ≤ 0.05, *; *p* ≤ 0.01, **; *p* ≤ 0.001, ***; *p* ≤ 0.0001, ****). CLL/N^WT^ CLL/*NOTCH1* WILD TYPE, CLL/N^M^ CLL/*NOTCH1* MUTATED.

Our previous data indicate that IgM cross-linking increases NOTCH1 protein levels and that BCR-induced calcium flux promotes ligand-independent NOTCH1 activation [21]. We selected a cohort of 64 primary CLL samples from untreated patients all with an unmutated BCR background and stratified as *NOTCH1*-mutated (CLL/N^M^) or *NOTCH1*-wild-type (CLL/N^WT^). BCR cross-linking led to activation of NOTCH1 signaling, which was significantly higher in CLL/N^M^ cells compared to CLL/N^WT^, as evidenced by western blot analysis (Fig. 1A). Expression levels of canonical *NOTCH1* target genes, such as *NRARP* and *DTX1* further confirmed these findings, with EDTA used as positive control to induce ligand-independent NOTCH1 activation (Fig. 1B). These results indicate that BCR stimulation alone is sufficient to enhance *NOTCH1* transcriptional activity, further supporting a functional crosstalk between the two pathways. RNA-seq data from a randomly down-sampled (balanced) cohort of CLL/N^WT^ (n = 50) and CLL/N^M^ (n = 50) [33] confirmed that NOTCH1 signaling pathway was significantly enriched in CLL/N^M^ cells (NES = 1.52, FDR = 0.106, Fig. 1C). Gene Set Enrichment Analysis (GSEA) also revealed enrichment of metabolic and proliferative pathways specifically in the CLL/N^M^ subset. Main categories included glycolysis (NES = 1.51, FDR = 0.006), mitochondrial biogenesis (NES = 1.90, FDR < 0.001), oxidative phosphorylation (NES = 1.32, FDR = 0.144), and reactive oxygen species (NES = 1.61, FDR = 0.109). Cell cycle-associated gene sets, such as mitotic spindle formation (NES = 1.64, FDR = 0.128) were also significantly enriched (Fig. 1C, Table S4-5). A pathway-level overview of the full MSigDB Hallmark collection is provided in Supplementary Fig. 1 (Table S4), placing NOTCH1 signaling and metabolic pathways in the context of all enriched signatures. Consistently, GO enrichment analysis of genes up-regulated in CLL/N^M^ highlighted mitochondrial respiratory/energy metabolism-related processes (Supplementary Fig. 1B). Differential expression results are summarized in Supplementary Fig. 1C (volcano plot, with representative leading-edge genes from GSEA annotated). Altogether, these results indicate that metabolic pathways are prominently enriched in CLL/N^M^ samples.

### Primary CLL/N^M^ cells show enhanced metabolic performances

**Fig. 2:**
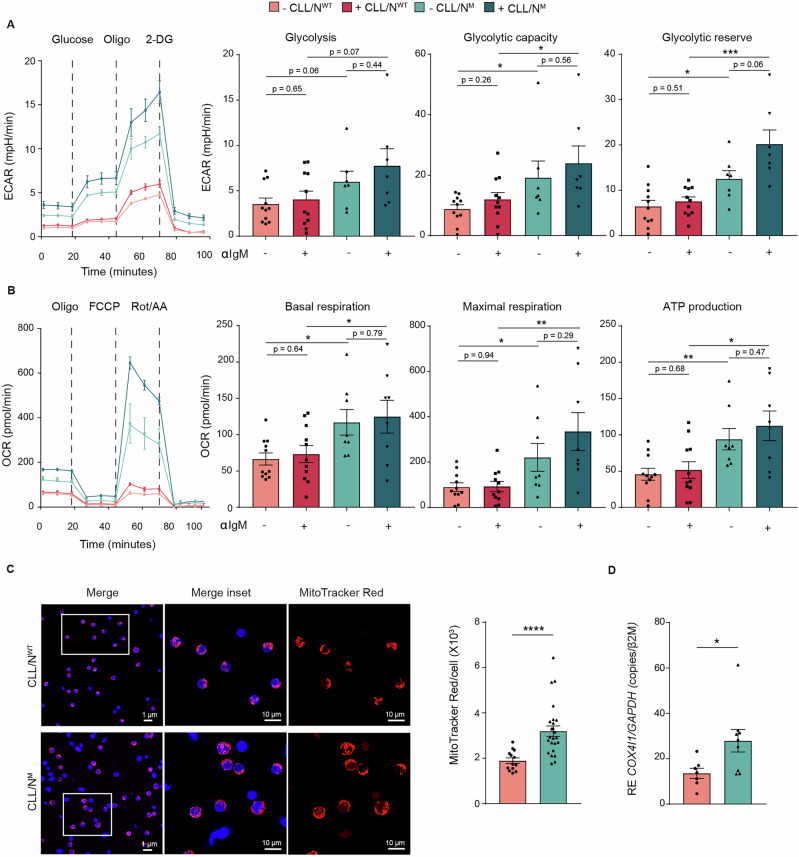
CLL/N^M^ cells show higher rates of glycolysis and mitochondrial respiration compared to CLL/N^WT^ cells. **A** Seahorse Glycolysis Stress Test measuring the extracellular acidification rate (ECAR) in CLL patients (CLL/N^WT^ *n* = 11, CLL/N^M^ *n* = 7) following the sequential addition of Glucose, Oligomycin (Oligo), and 2-deoxy-D-glucose (2-DG). A representative ECAR profile chosen from two different CLL patients is shown (CLL/N^WT^-pink and CLL/N^M^-blue, left panel). Bar graphs display quantification of glycolysis, glycolytic capacity, and glycolytic reserve under unstimulated (–) or stimulated (+) with plate-bound α-IgM for 24 h (right panel). **B** Seahorse Cell MitoStress Test measuring the oxygen consumption rate (OCR) in CLL patients (CLL/N^WT^ *n* = 11, CLL/N^M^ *n* = 8) following the sequential addition of inhibitors of mitochondrial function: Oligomycin (Oligo), Carbonyl cyanide p-trifluoromethoxyphenylhydrazone (FCCP), and a combination of antimycin A and rotenone (Rot/AA). A representative OCR profile chosen from two different CLL patients is shown (CLL/N^WT^-pink and CLL/N^M^-blue, left panel). Bar graphs graphs display quantification of basal respiration, maximal respiration, ATP production and spare respiratory capacity under unstimulated (–) or stimulated (+) with plate-bound α-IgM for 24 h (right panel). Basal respiration was calculated after subtraction of non-mitochondrial respiration. ATP-linked respiration was calculated following the addition of oligomycin. Maximal respiration was measured following the addition of FCCP. Spare respiratory capacity was calculated based on the difference between the basal respiration and maximal respiration. **C** Confocal microscopy images of CLL/N^WT^ and CLL/N^M^ cells. Merged images show nuclei stained in blue with Hoechst 33342 and mitochondria stained in red with MitoTracker Deep Red FM. Merge insets highlight representative areas of the merged signal. Mitochondrial signal alone (MitoTracker Deep Red FM) is also shown. Quantitative assessment of MitoTracker Deep Red FM fluorescence is also shown (right panel). **D** qRT-PCR analysis of *COX4I1*/*GAPDH* ratio in primary CLL cells (CLL/N^WT^ *n* = 7 and CLL/N^M^ *n* = 9) in basal conditions. mRNA expression was normalized over β-2-microglobulin (*β2M*). (*p* ≤ 0.05, *; *p* ≤ 0.01, **; *p* ≤ 0.001, ***; *p* ≤ 0.0001, ****).

To validate GSEA findings, we employed the Seahorse metabolic analyzer to measure both glycolysis and mitochondrial respiration, using the extracellular acidification rate (ECAR) and the oxygen consumption rate (OCR) as respective readouts. CLL/N^M^ cells were characterized by elevated ECAR compared to CLL/N^WT^ together with a low lactate production, suggesting an enhanced glycolytic flux directed toward mitochondrial metabolism (Fig. 2A and Supplementary Fig. 2A-B). Consistently, CLL/N^M^ cells displayed significantly higher OCR and ATP synthesis compared to CLL/N^WT^ (Fig. 2B). This was supported by increased proton leak and higher spare respiratory capacity associated with CLL/N^M^ cells, indicating highly active mitochondria capable of handling increased energy demands (Supplementary Fig. 2C-D). Notably, at baseline, CLL/N^M^ cells consistently displayed higher ECAR and OCR values, indicating enhanced metabolic activity even in standard conditions. Upon BCR stimulation, both ECAR and OCR further increased in CLL/N^M^ cells, resulting in a more pronounced metabolic activation. In line with these findings, CLL/N^M^ cells also displayed increased concentration of free mitochondrial radicals, as detected by MitoSOX Red staining, suggesting the presence of increased oxidative stress (Supplementary Fig. 2E). We then assessed mitochondrial density by staining with both MitoTracker Green and Red. The former was used in flow cytometry to stain total mitochondria irrespective of membrane potential, while the latter was used in confocal microscopy to visualize active, membrane potential-dependent mitochondria. Results showed that the increased aerobic mitochondrial metabolism in CLL/N^M^ cells was associated with higher mitochondrial density (Fig. 2C, D and Supplementary Fig. 2F). Notably, an extensive mitochondrial network was observed in CLL/N^M^ cells, compared to CLL/N^WT^, suggesting a correlation between this gene mutation and increased mitochondrial mass (Fig. 2C and Supplementary Fig. 2F). Consistently, we also observed an increase in mitochondrial DNA (mtDNA) content by quantifying the ratio of COX4I1 mRNA, a mitochondrial gene, to GAPDH mRNA, a nuclear gene, indicating a higher mitochondrial mass (Fig. 2D). Collectively, these results suggest that CLL/N^M^ are specifically sensitive to metabolic reprogramming driven by BCR signaling.

### Establishment and characterization of MEC-1/N^M^ and MEC-1/N^WT^ MEC-1 models recapitulating CLL metabolic signatures

To overcome CLL cell constraints, we developed a cellular model carrying unmutated BCR with or without *NOTCH1* mutations. We took advantage of the MEC-1 CLL-like cell line, which harbors a subpopulation of naturally occurring surface IgM-negative cells (Supplementary Fig. 3A-B). These cells, which express the mRNA for IGHV genes, but fail to express the surface protein, were cloned to obtain a homogeneous sIgM^-^ population and edited to obtain clones with truncating mutations in exon 34 to delete the *NOTCH1* PEST domain [21]. The resulting subclones were lentivirally transduced with an unmutated IGHV4-59-derived IgM heavy chain, cloned from a patient with UM-CLL (Supplementary Fig. 3C). Clones carrying robust and comparable surface IgM levels, with or without *NOTCH1* mutation (abbreviated as MEC-1/N^WT^ and MEC-1/N^M^) were then selected for functional assays (Supplementary Fig. 3D). To assess BCR functionality in the reconstituted MEC-1 models, we stimulated MEC-1/N^WT^ and MEC-1/N^M^ cells with soluble anti-IgM and observed phosphorylation of BTK and ERK1/2, indicating that *NOTCH1* mutations do not directly impact short-term BCR signaling (Supplementary Fig. 4A-B). Long-term activation with plate-bound α-IgM led to increased and comparable expression of *MYC* and *CCL3* in both cell lines, confirming intact BCR responsiveness regardless of *NOTCH1* status (Supplementary Fig. 4C). In addition, MEC-1/N^M^ cells showed higher expression levels of *NRARP* compared to MEC-1/N^WT^ cells, consistent with constitutive NOTCH1 signaling. Upon stimulation with either α-IgM or EDTA, *NRARP* expression further increased in both cells, but remained significantly higher in MEC-1/N^M^ cells, confirming the hyperactive NOTCH1 signaling status (Supplementary Fig. 4D). RNA-seq profiling confirmed that MEC-1/N^M^ cells recapitulate the metabolic and proliferative transcriptional programs identified in unstimulated primary CLL/N^M^ samples and demonstrated that these pathways are further reinforced upon 24-h α-IgM stimulation in the MEC-1 model (Supplementary Fig. S5-6). Principal component analysis (PCA) of the 20,395 protein-coding genes showed that MEC-1 cells samples cluster primarily by *NOTCH1* mutational status. For completeness, all pairwise principal component projections are reported in Supplementary Fig. S5. Supplementary Fig. S6A highlights the PC1 vs PC4 projection, and PCA restricted to genes in the NOTCH1 signaling pathway similarly segregated samples by *NOTCH1* status (Supplementary Fig. S6B).

Consistent with the patient-derived data, enrichment in metabolic and proliferative pathways was found in MEC-1/N^M^ cells in both basal and stimulated conditions (Supplementary Fig. 6C-D and Table S7). These observations were further supported by (Metabolic Reaction Enrichment Analysis) MaREA analysis, showing selective upregulation of key enzymatic programs, confirming *NOTCH1-*dependent metabolic reprogramming (Supplementary Fig. 7). Thus, this model recapitulates *NOTCH1* activity and CLL metabolic features, offering a valuable in vitro system.

### MEC-1/N^M^ cells are metabolically more active compared to MEC-1/N^WT^ cells

**Fig. 3:**
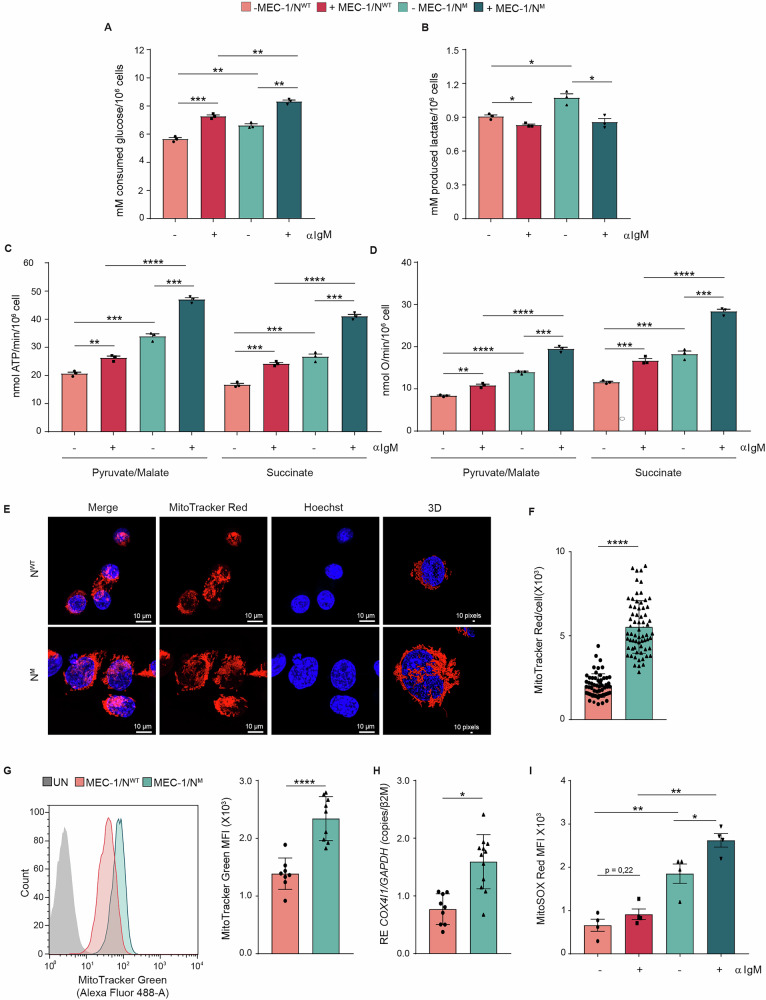
MEC-1/N^M^ cells display higher metabolism compared to MEC-1/N^WT^ cells. **A** Glucose consumption by MEC-1/N^WT^ and MEC-1/N^M^ cells either unstimulated (–) or stimulated (+) with plate-bound α-IgM for 24 h (*n* = 3). **B** Lactate concentration in the culture medium of MEC-1/N^WT^ and MEC-1/N^M^ cells either not treated (–) or stimulated (+) with plate-bound α-IgM for 24 h (*n* = 3). **C** ATP synthesis rate by MEC-1/N^WT^ and MEC-1/N^M^ cells either unstimulated (–) or stimulated (+) with plate-bound α-IgM for 24 h (*n* = 3). ATP synthesis coming from Complexes I, III, IV was measured with pyruvate/malate supplementation. ATP synthesis coming from Complexes II, III, IV was measured with succinate supplementation. **D** Oxygen consumption rate (OCR) of MEC-1/N^WT^ and MEC-1/N^M^ cells either unstimulated (–) or stimulated (+) with plate-bound α-IgM for 24 h (*n* = 3). Pyruvate/Malate and Succinate were provided as substrates as described in the ATP synthesis part. **E** Confocal microscopy images of MEC-1/N^WT^ and MEC-1/N^M^ cells. Nuclei are stained blue with Hoechst 33,342, MitoTracker Deep Red FM was used to stain mitochondria. Mitochondrial morphology was visualized by 3D reconstruction for confocal stacks. **F** Quantitative assessment of MitoTracker Deep Red FM fluorescence. **G** Representative flow cytometry profile of MitoTracker Green internalized by MEC-1/N^WT^ and MEC-1/N^M^ cells compared to unstained control (UN). MFI of MitoTracker Green internalized by unstimulated MEC-1/N^WT^ (*n* = 8) and MEC-1/N^M^ cells (*n* = 9) (right panel). **H** Determination of mtDNA/nDNA ratio as *COX4I1*/*GAPDH* by real-time PCR as an index of mitochondrial mass (MEC-1/N^WT^ *n* = 9 and MEC-1/N^M^ *n* = 12). mRNA expression was normalized over β-2-microglobulin (*β2M*). **I** MFI of MitoSOX Red internalized by MEC-1/N^WT^ (*n* = 4) and MEC-1/N^M^ (*n* = 4) cells either unstimulated (–) or stimulated (+) with aIgM for 24 h. (*p* ≤ 0.05, *; *p* ≤ 0.01, **; *p* ≤ 0.001, ***; *p* ≤ 0.0001, ****). MEC-1/N^WT^ MEC-1/*NOTCH1* WILD TYPE, MEC-1/N^M^ MEC-1/*NOTCH1* MUTATED.

We then used these clones to recapitulate the metabolic signature observed in primary CLL cells. Results showed a significantly higher glucose consumption in MEC-1/N^M^ compared to MEC-1/N^WT^ cells in basal conditions, which robustly increased upon BCR engagement, in line with previous findings (Fig. 3A) [34–36]. BCR activation caused a slight but significant reduction in lactate production, especially in MEC-1/N^M^ cells, suggesting that glucose may be differentially used to fuel the tricarboxylic acid (TCA) cycle rather than glycolysis (Fig. 3B). We then focused on the role of mitochondria in metabolic reprogramming. Rates of OCR, as well as ATP synthesis, were lower in MEC-1/N^WT^ cells than in the MEC-1/N^M^, a condition boosted after BCR ligation (Fig. 3C, D). These findings were further supported by analyses of mitochondrial content and organization, which revealed that the increased aerobic mitochondrial metabolism in MEC-1/N^M^ cells was associated with higher mitochondrial density. Notably, an extensive mitochondrial network was observed in MEC-1/N^M^ cells, compared to MEC-1/N^WT^, reinforcing the association between this *NOTCH1* mutations and increased in mitochondrial mass, consistent with findings in CLL/N^WT^ and CLL/N^M^ (Fig. 3E–G and Video 1-2). Accordingly, MEC-1/N^M^ cells displayed a significantly higher COX4I1/GAPDH ratio compared to MEC-1/N^WT^ clones (Fig. 3H). In addition, MEC-1/N^M^ cells exhibited higher free radical concentrations compared to MEC-1/N^WT^, both at baseline and after 24-h BCR stimulation (Fig. 3I). Altogether, these findings validate the metabolic reprogramming observed in primary CLL cells and confirm that this adaptation is associated to *NOTCH1* mutation.

### Stable isotope tracing analysis identifies glutamine as the main fuel for the energetic processes of MEC-1/N^M^ and CLL/N^M^ cells

**Fig. 4:**
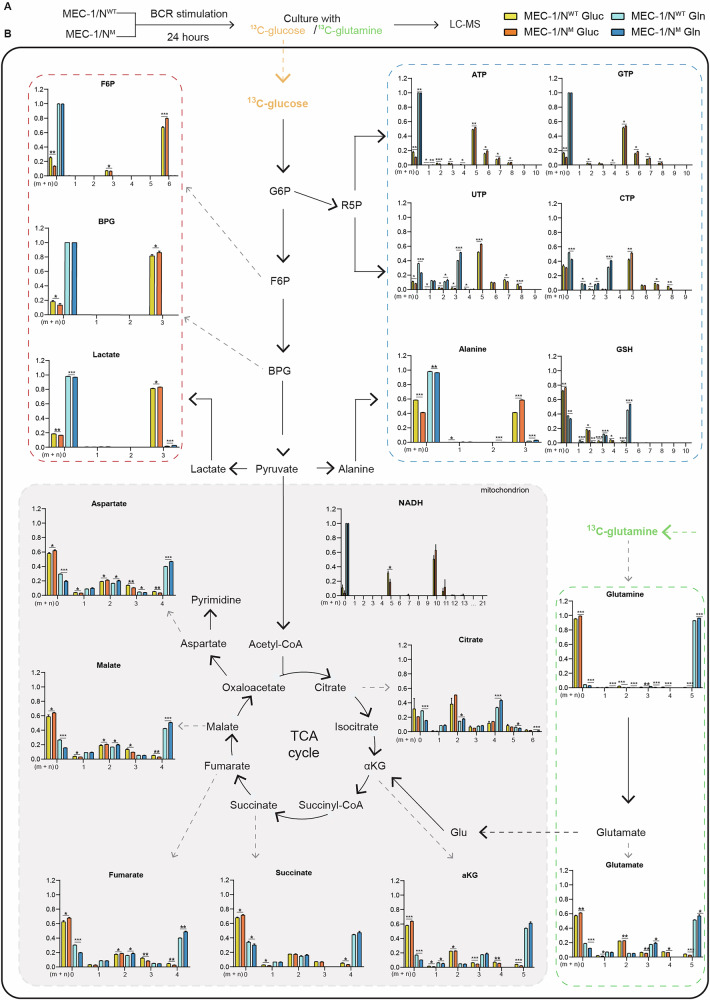
Glucose and glutamine fueling of BCR-stimulated MEC-1/N^WT^ and MEC-1/N^M^ cells. **A** Metabolomic isotopologue analysis was performed in MEC-1/N^WT^ and MEC-1/N^M^ cells stimulated with plate-bound α-IgM for 24 h and subsequently cultured in medium containing either [U-^13^C]-glucose (orange) and [U-^13^C]-glutamine (green) (*n* = 3). Incorporation of ^13^C in metabolic intermediates was analyzed by liquid chromatography–mass spectrometry (LC-MS). **B** Mass isotopologue distributions (MID) of each m + n isotopologue were plotted to assess ¹³C incorporation from fully labeled glucose (MEC-1/N^WT^, yellow; MEC-1/N^M^, orange) or glutamine (MEC-1/N^WT^, light blue; MEC-1/N^M^, dark blue) into selected metabolites following BCR stimulation. The red dashed panel indicates the glycolytic/fermentative arm (F6P, BPG, and lactate). The blue dashed panel compiles anabolic pathways, including de novo nucleotide biosynthesis (ATP, GTP, UTP, CTP) and amino acid/antioxidant pools (alanine, GSH). The green dashed panel depicts glutaminolysis (glutamine and glutamate). Data represent mean ± SD. (*p* ≤ 0.05, *; *p* ≤ 0.01, **; *p* ≤ 0.001, ***; *p* ≤ 0.0001, ****). F6P fructose-6-phosphate, BPG bisphosphoglycerate, GSH reduced glutathione.

To investigate whether enhanced mitochondrial metabolism observed in MEC-1/N^M^ was primarily fueled by glucose or glutamine, we performed stable isotope tracing on MEC-1/N^WT^ and MEC-1/N^M^ cells cultured for 24 h with plate-bound αIgM in media containing either [U-^13^C]-glucose or [U-^13^C]-glutamine (Fig. 4A). We then analyzed metabolites from pentose phosphate pathway (PPP), nucleotide and amino acid metabolism, and the TCA cycle. Mass isotopologue distributions (MIDs), representing the incorporation of ^13^C-labeled substrates into metabolites (from m + 0 to m + n, where n is the maximum number of carbon atoms), revealed a higher incorporation of ^13^C from [U-^13^C]-glucose in all the glycolytic intermediates of MEC-1/N^M^ cells, confirming that *NOTCH1* hyperactivation increases glycolytic flux. Importantly, incorporation of ^13^C from [U-^13^C]-glucose was greater in MEC-1/N^M^ cells, possibly linked to increased nucleotide synthesis and the generation of reducing equivalents to support proliferation. In contrast, glucose-derived labeling was significantly higher in MEC-1/N^WT^ cells across nearly all TCA cycle intermediates, while MEC-1/N^M^ cells displayed significantly higher levels of glutamine-derived labeling in all TCA intermediates. Labeling from [U-^13^C]-glutamine was also significantly enriched in glutathione and aspartate in MEC-1/N^M^ cells, suggesting the involvement of glutamine not only as an energy substrate but also as a carbon source for biosynthetic processes (Fig. 4B). Stable isotope tracing was also replicated in CLL/N^WT^ and CLL/N^M^ cells and under the same experimental conditions (Supplementary Fig. 8A). MIDs showed generally reduced tracer incorporation overall, as observed by the higher proportion of m + 0 (Supplementary Fig. 8B). This is consistent with the fact that primary CLL cells are characterized by low proliferative rates in vitro. Nonetheless, labeling patterns for multiple TCA cycle intermediates (including aspartate, malate, fumarate, citrate, and α-ketoglutarate) mirrored those observed in the MEC-1/N^M^ cells, with higher glutamine-derived incorporation in CLL/N^M^ compared to CLL/N^WT^ (Supplementary Fig. 8B). We next assessed glucose and glutamine uptake: MEC-1/N^M^ and CLL/N^M^ cells displayed significantly higher glutamine uptake compared to their counterparts, aligning with the isotope tracing data and indicating increased glutamine fueling of the TCA cycle (Supplementary Fig. 9A). Glucose uptake was also enhanced in MEC-1/N^M^ and CLL/N^M^ cells, mirroring the glycolytic profile observed (Supplementary Fig. 9B). Together, these data suggest that glutamine serves as the primary metabolic fuel sustaining TCA cycle and mitochondrial metabolism in *NOTCH1-*mutated cells.

### Metabolic reprogramming due to *TFAM* transcriptional regulation in MEC-1/N^M^ cells

**Fig. 5:**
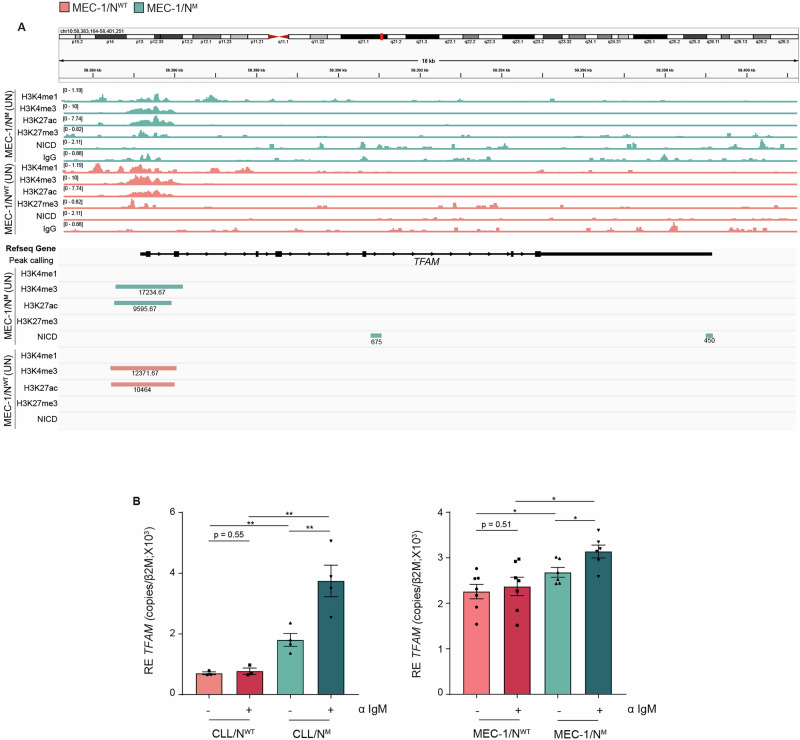
*NOTCH1*-Mediated Transcriptional Regulation of *TFAM.* **A** Genome browser tracks (visualized in IGV) showing CUT&Tag data for the NICD domain of NOTCH1 at the TFAM locus in wild-type (MEC-1/N^WT^) and *NOTCH1*-mutant (MEC-1/N^M^) cells. Each track represents the merged signal from three biological replicates (*n* = 3) of untreated MEC-1/N^WT^ and MEC-1/N^M^ samples. Histone modifications (H3K4me1, H3K4me3, H3K27ac, H3K27me3) and IgG (as control) are also shown to provide chromatin context. The lower panels display peaks called with SEACR for each histone mark and NICD, highlighting regions of significant enrichment. Numbers indicate the value of intensity of the peaks. **B** qRT-PCR analysis of *TFAM* expression in both CLL/N^WT^ (*n* = 3) and CLL/N^M^ (*n* = 4) cells (left panel) and MEC-1/N^WT^ (*n* = 7) and MEC-1/N^M^ (*n* = 6) cells (right panel) under basal (–) or aIgM stimulated (+) conditions. mRNA expression was normalized over β-2-microglobulin (*β2M*). (*p* ≤ 0.05, *; *p* x ≤ 0.01, **; *p* ≤ 0.001, ***; *p* ≤ 0.0001, ****).

To elucidate the mechanisms underlying metabolic reprogramming in *NOTCH1*-mutated cells, we performed high-throughput CUT&Tag profiling. While the analysis was unbiased, we focused on pathways related to metabolism. Differential binding analysis of the activating histone mark H3K4me3 (associated with transcription start sites) revealed significant enrichment in MEC-1/N^M^ cells at genes associated with mitochondrial transcription and translation, in both untreated (UN) and BCR-stimulated (IgM) conditions (TableS6-7). In the UN condition, gene ontology (GO) enrichment included Mitochondrial RNA 3’-End Processing (GO:0000965), Ribosome Disassembly (GO:0032790), and Mitochondrial Translation (GO:0032543). Key genes involved in these processes, such as *TRMT10C*, *MTIF3*, and mitochondrial ribosomal protein genes (*MRPSs*, *MRPLs*), showed increased H3K4me3 signal, suggesting transcriptional activation of the mitochondrial gene expression machinery in MEC-1/N^M^ cells (Table S6). These results point to a broader transcriptional up-regulation of the mitochondrial functional network in MEC-1/N^M^ cells. Upon BCR cross-linking, the enriched GO terms included Mitochondrial Genome Maintenance (GO:0000002) and Regulation of Establishment of Protein Localization to Mitochondrion (GO:1903747), indicating a sustained engagement of mitochondrial gene regulatory programs in MEC-1N^M^ cells (Table S7). Given the up-regulation of several genes involved in mitochondrial transcription and translation, we investigated Mitochondrial Transcription Factor A (*TFAM*), a nuclear-encoded transcription factor essential for mtDNA transcription and replication [37], as a potential master regulator driving this program. DBA analysis revealed significant gain of the active transcription mark H3K27Ac at the *TFAM* promoter in MEC-1/N^M^ cells compared to MEC-1/N^WT^ (log₂FC = 0.47, FDR = 0.007), with no differential enrichment for the repressive histone mark H3K27me3 (Fig. 5A). Notably, CUT&Tag for NICD revealed clear binding at the TFAM locus in MEC-1/N^M^ cells under both UN and IgM-stimulated conditions, with no binding detected in MEC-1/N^WT^ cells (Fig. 5A, Supplementary Fig. 10A). RNA-seq data and qRT-PCR analyses revealed higher levels of *TFAM* expression in MEC-1/N^M^ and CLL/N^M^ cells, compared to their counterpart, in line with *NOTCH1*-mediated positive regulation of the *TFAM* locus (Fig. 5B, Supplementary Fig. 10B).

### MEC-1/N^M^ CLL cells rely on TFAM for mitochondrial integrity and survival

**Fig. 6:**
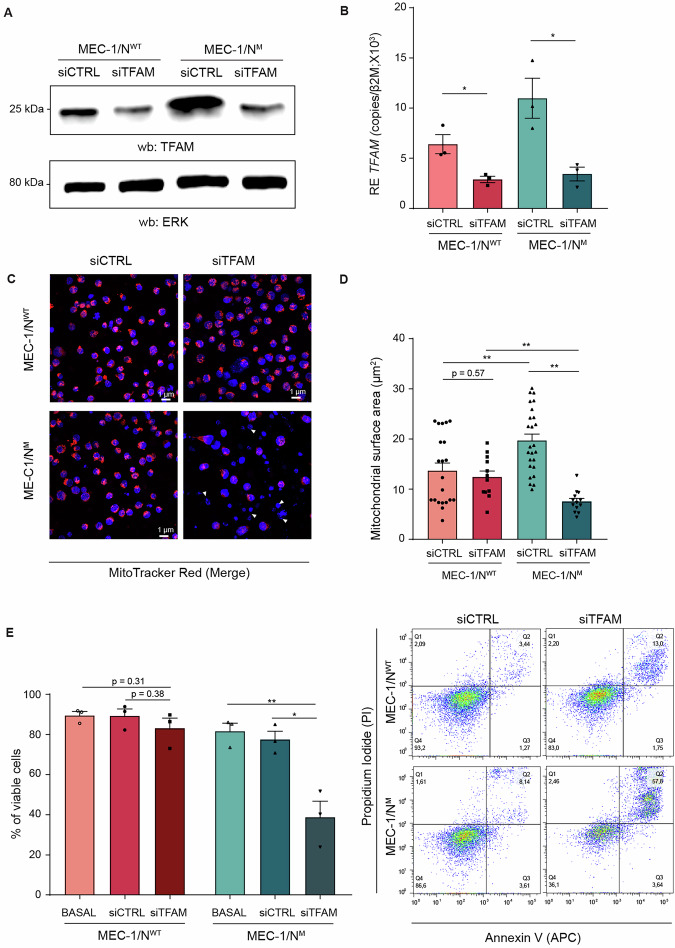
*TFAM* knockdown reveals TFAM dependency of *NOTCH1*-mutated CLL cells in mitochondrial regulatory circuitry. **A** Western blot analyses of TFAM protein’s steady-state levels in MEC-1/N^WT^ and MEC-1/N^M^ cells in the presence of small interfering RNAs (siRNAs) against controls (siCTRL) and *TFAM* (siTFAM). Images are representative of *n* = 3 independent experiments. ERK was used as a loading control. **B** qtRT-PCR analysis of *TFAM* expression in MEC-1/N^WT^ and MEC-1/N^M^ cells in the presence of siCTRL and siTFAM. mRNA expression was normalized over β-2-microglobulin (*β2M*). **C** Representative confocal microscopy images of MEC-1/N^WT^ and MEC-1/N^M^ cells upon *TFAM* silencing, stained with MitoTracker Deep Red FM to visualize mitochondria and Hoechst 33342 (blue) for nuclei. Merged channel images are shown. White arrows indicate dying/dead cells in the MEC-1/N^M^-siTFAM condition. **D** Quantification of mitochondrial surface area from multiple independent images of MEC-1/N^WT^ and MEC-1/N^M^ cells following *TFAM* knockdown. **E** Percentage of viable MEC-1/N^WT^ and MEC-1/N^M^ cells assessed by Annexin V/7-AAD staining in basal conditions, siCTRL, and following *TFAM* knockdown (siTFAM, left). Representative dot plots of FC upon *TFAM* silencing (right). (*p* ≤ 0.05, *; *p* ≤ 0.01, **; *p* ≤ 0.001, ***; *p* ≤ 0.0001, ****).

Based on epigenomic and transcriptional data implicating TFAM as a potential master regulator of the overall metabolic reprogramming, we tested functional dependence of MEC-1/N^M^ cells on TFAM. Partial knockdown of TFAM using siRNA in MEC-1/N^WT^ and MEC-1/N^M^ cells, confirmed by Western blot and qRT-PCR (Fig. 6A, B and Supplementary Fig. 10C), led to a decrease in mitochondrial mass, selectively in MEC-1/N^M^ cells while in MEC-1/N^WT^ it remained unaffected (Fig. 6C and Supplementary Fig. 10D). This difference was confirmed by quantification of both mitochondrial surface area and volume (Fig. 6D, Supplementary Fig. 10E), which were significantly decreased in MEC-1/N^M^ cells upon TFAM loss but remained unaffected in MEC-1/N^WT^ cells. Confocal images also revealed a higher proportion of apoptotic MEC-1/N^M^ cells following *TFAM* silencing, as indicated by the absence of MitoTracker staining and the presence of condensed Hoechst-positive nuclei (Fig. 6C). Annexin V/PI staining of silenced cells demonstrated a significant increase in apoptosis specifically in MEC-1/N^M^ cells after *TFAM* knockdown, while MEC-1/N^WT^ cells remained viable (Fig. 6E). Altogether, these results demonstrate that MEC-1/N^M^ cells are uniquely dependent on TFAM for mitochondrial maintenance and survival, underscoring *TFAM* as a critical node in the metabolic reprogramming driven by *NOTCH1* mutations.

### *NOTCH1*-mutated cells rely on glutamine metabolism to sustain their proliferative advantage and show enhanced sensitivity to glutamine inhibition combined with venetoclax

**Fig. 7:**
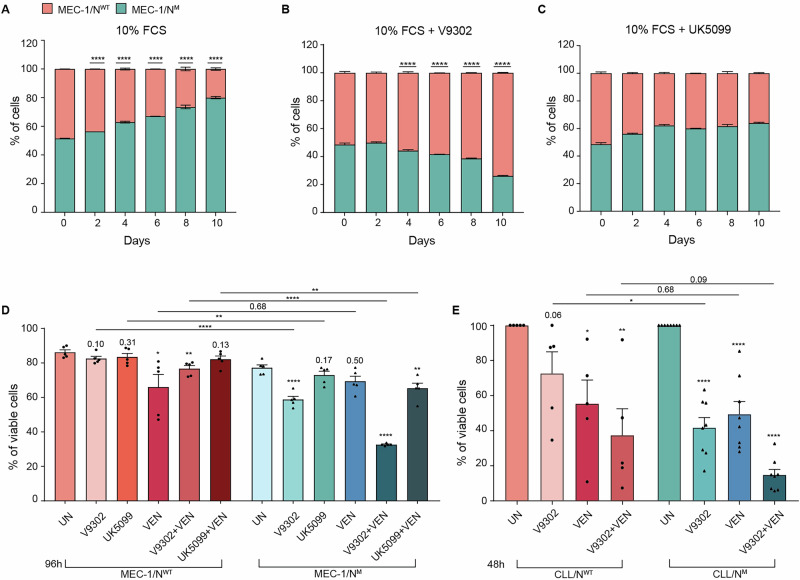
*NOTCH1*-mutated CLL cells show increased sensitivity to glutamine blockade and Venetoclax combination treatment. Cell viability analyses determined by AV/PI staining in MEC-1/N^WT^ and MEC-1/N^M^ cells co-cultured in standard condition (RPMI + 10% FCS) for 10 days (**A**), in the presence of the glutamine transporter ASCT2 Inhibitor V-9302 (**B**), or in the presence the mitochondrial pyruvate carrier inhibitor UK-5099 (**C**). Asterisks refer to the significance compared to day 0. **D** Percentage of viable MEC-1/N^WT^ and MEC-1/N^M^ cells, after the following conditions: untreated (UN), V-9302, UK-5099, venetoclax (VEN), V-9302 + venetoclax (V-9302 + VEN), and UK-5099 + venetoclax (UK5099 + VEN), for 96 h (*n* = 3). **E** Percentage of viable CLL/N^WT^ and CLL/N^M^ cells (CLL/N^WT^ *n* = 5 and CLL/N^M^ *n* = 8) after the following conditions: UN, V-9302, VEN and V-9302 + VEN, for 48 h. Asterisks and values on the columns indicate the significance and corresponding *p* values compared to the UN condition (*p* ≤ 0.05, *; *p* ≤ 0.01, **; *p* ≤ 0.001, ***; *p* ≤ 0.0001, ****).

Given that metabolic reprogramming is associated with increased tumor survival, proliferation, and aggressiveness [18, 38], we focused on investigating whether an elevated metabolic profile correlated with a growth advantage. To the purpose, MEC-1/N^M^ (GFP-labeled) and MEC-1/N^WT^ (RFP-labeled) cells were co-cultured (1:1) for 10 days and cell proliferation kinetics assessed to monitor their relative expansion under identical conditions. When cells were cultured in standard condition, MEC-1/N^M^ cells gradually outgrew the counterpart, becoming the dominant population (Fig. 7A) and indicating a clear advantage conferred by the *NOTCH1* mutation. We next investigated whether this proliferative advantage was dependent on the availability of glucose and glutamine. To this end, we tested the effect of selective metabolic inhibitors UK5099 (mitochondrial pyruvate carrier inhibitor) [39] V-9302 (inhibitor of the glutamine transporter ASCT2) [40, 41] V-9302 reversed the competitive outgrowth of MEC-1/N^M^ cells, resulting in a relative enrichment of MEC-1/N^WT^ cells over time (Fig. 7B). In contrast, blocking glucose-derived oxidative metabolism, using UK5099 did not significantly affect co-culture kinetics (Fig. 7C), underscoring the predominant role of glutamine over glucose in sustaining the competitive fitness advantage of MEC-1/N^M^ cells.

In apoptosis assays performed in MEC-1/N^M^ cells and CLL/N^M^ samples, glutamine inhibition with V-9302 significantly reduced cell viability compared to the untreated condition, confirming a metabolic dependency on glutamine (Fig. 7D, E). In contrast, glucose inhibition had a minimal effect on cell viability in both cases and in line with the proliferation assays.

Lastly, to assess whether this metabolic dependency could become a therapeutic vulnerability, we decided to test cells with the BCL-2 inhibitor venetoclax as an inducer of mitochondrial apoptosis. MEC-1/N^M^ and CLL/N^M^ cells were constitutively more sensitive to venetoclax compared to their counterparts, whereas the combination of V-9302 and venetoclax led to a further significant reduction in viability exclusively in *NOTCH1*-mutated subsets (Fig. 7D). The two agents showed synergic action, as determined by the combination index (Supplementary Fig. 11A). This effect was not observed when venetoclax was combined with glucose inhibition, reinforcing the idea that glutamine, but not glucose, is the critical metabolic vulnerability in CLL/N^M^, and suggesting that targeting this dependency can sensitize cells to venetoclax (Fig. 7D, E and Supplementary Fig. 11B-C).

## Discussion

Our study uncovers how *NOTCH1* mutations reprogram mitochondrial metabolism in CLL, establishing a mechanistic connection between oncogenic signaling and metabolic plasticity. *NOTCH1* is recurrently mutated in CLL patients, and the prevalence of its mutations increases with disease progression, arguing in favor of a direct role of the protein in this process. Building on previous evidence of BCR-*NOTCH1* crosstalk [21], we investigated whether this signaling axis could shape the metabolic phenotype of CLL cells. In a cohort of BCR-UM samples, BCR engagement triggered NOTCH1 activation in both wild-type and mutated backgrounds, but with CLL/N^M^ exhibiting more sustained responses. NOTCH1 activation was confirmed by EDTA treatment, providing a rationale to focus our investigations solely on BCR stimulation (Fig. 1). Given the limited proliferative capacity and spontaneous in vitro mitochondrial apoptosis of primary CLL cells, we developed an isogenic MEC-1 model harboring either wild-type or mutated *NOTCH1* in an UM-BCR background (Supplementary Fig. 2). Through integrated transcriptomic and metabolic analyses, we found that *NOTCH1*-mutated cells exhibit a distinct bioenergetic profile, characterized by upregulation of mitochondrial programs, enhanced oxidative phosphorylation, and elevated ROS production, all hallmarks of an active mitochondrial phenotype (Figs. 2–4). Stable isotope tracing revealed a dual metabolic strategy in which glucose is redirected to the pentose phosphate pathway (PPP), while glutamine fuels the TCA cycle, highlighting a selective reliance on glutamine to sustain mitochondrial output [42]. This preferential use of glutamine was especially evident in MEC-1/N^M^ cells, which displayed significantly higher uptake rates, corroborating recent findings and reinforcing the centrality of this amino acid in *NOTCH1*-driven metabolic reprogramming [41–45]. Consistently, metabolomics in primary CLL samples showed that glutamine, rather than glucose, predominantly supports mitochondrial function in MEC-1/N^M^ cases, suggesting a shift away from glucose-driven biosynthesis (Fig. 4 and Supplementary Fig. 6). This dual activation of PPP and glutaminolysis not only fuels mitochondrial respiration but also sustains proliferation, conferring a growth advantage tightly linked to *NOTCH1* mutational status and consistent with the transcriptomic signature.

The breakthrough finding of our study is the identification of TFAM, a master regulator of mitochondrial transcription and replication [37], as a direct target of *NOTCH1*. Using epigenomic profiling, we demonstrate that the NICD directly binds and activates the TFAM locus, leading to increased expression of this mitochondrial factor and elevated mtDNA content (Fig. 5 and Supplementary Fig. 8A). *TFAM* knockdown selectively disrupted mitochondrial structure and viability in MEC-1/N^M^ cells, with no effect in the MEC-1/N^WT^ counterparts, establishing TFAM as a critical downstream effector of *NOTCH1*-dependent metabolic remodeling (Fig. 6 and Supplementary Fig. 8B–E). Functionally, this reprogramming translated into increased proliferation. These findings are consistent with recent in vivo studies supporting a role for sustained *NOTCH1* activation in promoting disease evolution [46, 47]. The second relevant finding of our study is the selective dependence on glutamine of MEC-1/N^M^ cells which can be exploited therapeutically as previously shown for CLL cells [41, 48]. Consistently, our results show that glutamine transport blockade primes MEC-1 cells, as well as primary CLL cells to the effect of BCL-2 inhibitor venetoclax, with the two drugs showing synergic action (Supplementary Fig. 9A). It is important to stress that, this effect is also seen in the MEC-1 cells, which are typically resistant to venetoclax action due to their *TP53* mutation (Fig. 7) [49].

In conclusion, our work shows that BCR ligation in *NOTCH1*-mutated CLL cells induces metabolic adaptation, with increased mitochondrial activity, mediated through the activation of TFAM transcription factor. This axis uncovers an actionable metabolic dependency on glutamine, specific for *NOTCH1* mutated subset. The translational inference is that CLL cells are sensitive to glutamine import blockade. However, as tumor cells tend to adapt to pharmacological inhibition of a single pathway, we reasoned that combinations would be most successful. Accordingly, in our study MEC-1/N^M^ cells showed maximal responses to BCL-2 inhibition in the presence of V-9302. Combinatorial strategies of metabolic inhibitors and apoptotic inducers are rapidly emerging in hematological cancers: for example, CB-839 (telaglenastat), a selective GLS1 inhibitor, has shown potent synergy with BCL2 inhibition in lymphoma and AML models by increasing oxidative stress and collapsing mitochondrial respiration [50, 51]. In parallel, pegcrisantaspase (PegC), an amino acid–depleting enzyme that lowers circulating glutamine and asparagine, demonstrated clinical activity when combined with venetoclax in relapsed/refractory AML [52], paving the way for future clinical trials. These and other data support the concept that therapeutically targeting glutamine metabolism can dismantle mitochondrial resilience and restore apoptotic competence, providing a mechanistically grounded strategy to enhance venetoclax efficacy and overcome resistance in *NOTCH1*-mutated cells.

Our study also has some limitations. First, we used isolated primary CLL cells in the absence of their native microenvironment, nullifying the possibility of NOTCH1 being ligand activated and of multiple other cellular and soluble players. Second, we have used a highly artificial cellular model and highly selected primary cells, reducing the natural complexity of a disease characterized by multiple and co-existing mutations [53–55]. Future work will clarify the role of the microenvironment and of other molecular players in this novel axis and whether MYC cooperates with or amplifies *NOTCH1*-driven metabolic rewiring downstream of BCR engagement.

## Supplementary information

Supplemental material

Video 1 -MEC-1/ NOTCH1 WILD TYPE

Video 2 -MEC-1/ NOTCH1 MUTATED

## Data Availability

Data in this manuscript can be accessed via an email to the corresponding author. RNA-seq data is deposited in the EGA (European Genome-Phenome Archive) database (EGAD50000001435). All other raw data needed to evaluate the conclusions in the paper are present in the Supplementary Materials.
