# Supplementary material for: Functional cooperation between the B-cell receptor and NOTCH1 in regulating metabolic reprogramming in chronic lymphocytic leukemia
Source: Leukemia. 2026 Mar 23;40(5):982–95. doi: 10.1038/s41375-026-02912-7 (PMC13149317; doi:10.1038/s41375-026-02912-7)
Supplement: Supplementary file 1 — Supplemental material [file 41375_2026_2912_MOESM1_ESM.pdf]

SUPPLEMENTARY MATERIAL AND METHODS

Title: Functional cooperation between the BCR and *NOTCH1* in regulating metabolic reprogramming in chronic lymphocytic leukemia

Authors: Amelia Fascì[1], Francesco Edoardo Vallone[1], Nahal Nabelsi[1], Elodie Viry[2], Ilenia Sana[3], Alessia Morabito[4], Silvia Seghezzi[1], Noemi Anna Pesce[1], Matteo Rovere[1], Nadia Bertola[5], Chloé Duculty[2], Silvia Ravera[6], Samir Mouhssine[7], Marta Muzio[3], Paolo Ghia[3,4], Candida Vitale[8], Marta Coscia[8], Etienne Moussay[2], Gianluca Gaidano[7], John Allan[9], Richard R. Furman[9], Jerome Paggetti[2], Tiziana Vaisitti[1], Silvia Deaglio[1]

Affiliations: [1]Laboratory of Functional Genomics, Department of Medical Sciences, University of Turin, Turin, Italy; [2]Tumor Stroma Interactions, Department of Cancer Research, Luxembourg Institute of Health, Luxembourg, Luxembourg; [3]Division of experimental Oncology, IRCCS Ospedale San Raffaele, Milano, Italy; [4]Medical School, Università Vita-Salute San Raffaele, Milano, Italy; [5]IRCCS Ospedale Policlinico San Martino, Genoa, Italy; [6]Department of Experimental Medicine, University of Genoa and IRCCS Ospedale Policlinico San Martino, Genoa, Italy; [7]Division of Hematology, Department of Translational Medicine, University del Eastern Piedmont and Azienda Ospedaliero-Universitaria Maggiore della Carità, Novara, Italy; [8]Department of Molecular Biotechnology and Health Sciences, University of Turin, Turin, Italy; [9]Department of Hematology, Weill Cornell Medicine, New York, New York, USA.

Corresponding author: Silvia Deaglio, MD, PhD, Laboratory of Functional Genomics, Department of Medical Sciences, University of Turin, via Nizza 52, 10126 TORINO, Italy. Phone: (+39-011) 6709535. Email: silvia.deaglio@unito.it.

Data sharing statement: data in this manuscript can be accessed via an email to the corresponding author. RNA-seq data is deposited in the EGA (European Genome-Phenome Archive) database (EGAD50000001435).

Supplementary Material and Methods

Molecular cloning and lentivirus-mediated generation of MEC-1 clones

The unmutated IGHV sequence of the CLL #1 patient was amplified from the patient cDNA using the GoTaq Hot Start Polymerase (Promega) and the following primers:

FWD 5’ TTCTAGAGCCACCATGAAACATCTGTGGTTCTTCCTTCTCCTGGTG 3’

REV 5’ TGTCGACTCATTTCACCTTGAACAAGGTGACGGTGGT 3’

The amplification protocol was 95°C for 2 min., followed by 36 cycles each at 95ºC for 45 s, 1 min, at 57ºC, 2 min. 30 s at 72ºC, followed by a last elongation step at 72ºC for 10 min.

PCR products were purified from 1% agarose gels using the QIAquick Gel Extraction Kit (Qiagen, Hilden, Germany) and, after digestion with XbaI and SalI restriction enzymes (New England BioLabs, Ipswich, MA), they were ligated in the 3rd generation lentiviral construct pRRLSIN.cPPT.PGK-GFP.WPRE (Addgene, Watertown, MA), also digested with XbaI and SalI and purified from a 1% agarose gel, using the Quick Ligation Kit (New England BioLabs), according to the manufacturer's protocol. 3 µL of the ligation were then used to transform 50 L of DH5α competent E.coli (Subcloning Efficiency DH5α Competent Cells, Invitrogen, Waltham, MA). Bacteria were plated on LB-agar+ampicillin (100 µg/mL) and incubated at 37°C, single colonies were then inoculated in liquid LB+ampicillin medium and, after overnight (o.n.) incubated at 37ºC under shaking, plasmid DNA was purified with the QIAprep Spin Miniprep Kit (Qiagen). Plasmid DNA was screened by restriction digestion and/or PCR and the sequence was confirmed by Sanger sequencing (Eurofins Genomics. Luxembourg).

Lentiviral particles containing the unmutated *IGHV* under the hPGK constitutive promoter were generated by transfecting HEK293T cells with packaging and envelope plasmids (2nd generation lentiviral system) using Effectene (Qiagen), according to the manufacturer's

protocol. Viral particles were retrieved after 48 hours of culture using the Lenti-X Concentrator (Takara Bio, Kusatsu, Japan), resuspended in PBS, and stored at -80ºC. 10-30 µL of the concentrated lentiviral stock were used to transduce 70-80% confluent MEC-1/sIgM$^-$ cells. These were obtained from DSMZ (Braunschweig, Germany) and cultured according to the provider's recommendations. They were routinely screened for mycoplasma contamination and were consistently found to be mycoplasma-negative throughout the study. They were seeded in 6-well plates in a complete medium supplemented with 8 µg/mL of polybrene. After 24h, cells were split in a fresh medium. Single-cell clones of the infected bulk of MEC-1/sIgM$^-$ cells were obtained again by limiting dilution cloning, evaluated by flow cytometry (FC), and named MEC-1$^{IgM\text{-}UM}$N^{WT}. For *NOTCH1*-mutated cells, the CRISPR/Cas9-generated MEC-1/sIgM$^-$N^M clone was employed[1], which, similarly to MEC-1/sIgM$^-$, does not express sIgM. MEC-1$^{IgM\text{-}UM}$N^M clones were also generated as previously described. Red fluorescent protein (RFP) and green fluorescent protein (GFP) positive clones were generated by infecting cells with the same lentiviral construct carrying either RFP or GFP.

BCR ligation

The first protocol for BCR stimulation is short (1, 5, 10 minutes in suspension). Briefly, MEC-1 or primary CLL cells were starved in 0.1% FCS for 12 hours and then 1,5×10^6 cells were resuspended in ~80 µL PBS, kept on ice for 30-40 minutes, and stimulated with 5 µg of anti-IgM for 1, 5, or 10 minutes at 37°C. All samples, regardless of their stimulation protocol, were incubated together for 10 minutes at 37ºC for consistency. Stimulation was stopped by placing samples on ice and adding cold PBS. Cells were lysed right afterward.

The second protocol for BCR stimulation is long stimulation (6 hours-24 hours, anti-IgM-coated plate). Briefly, cells were starved in 0.1% FCS for 12 hours, then resuspended in a

complete medium, and finally incubated for the experiment-specific stimulation time (37°C, 5% CO_2) in a 24-well plate ($1{,}5\times10^6$ cells/well), either coated or not with anti-IgM. To coat the wells with anti-IgM, 24-well plates were incubated o.n. at 4ºC, with 10 µg of anti-IgM in 250 µL PBS per well. Plate-bound α-IgM was used to induce sustained signalling by preventing antibody internalization.

EDTA-mediated NOTCH1 activation

To induce NOTCH1 cleavage, ethylenediaminetetraacetic acid (EDTA, Sigma) was used either at 5mM for 15 minutes (short stimulation) or at 1mM for 6 hours (prolonged stimulation). MEC-1 or primary CLL cells were thawed and cultured in RPMI 10% FCS for 18 hours in the presence of 10mM DAPT to induce complete shutdown of NOTCH1 pathway. After washing away DAPT, in the case of short stimulation, cells were treated with 5mM EDTA for 15 minutes, then washed twice with PBS to remove residual EDTA. In the case of prolonged stimulation, cells were incubated for 6 hours with 1mM EDTA. Where indicated, 10mM DAPT was added to the cells during EDTA treatment as well as in the following hours of culture.

Flow Cytometry (FC)

5×10^5 cells were harvested and washed with 2 mL of FC buffer (9 g/L NaCl, 1 g/L BSA, 0.1% sodium azide). Samples were then resuspended in 100 µL of FC buffer containing the desired antibodies, incubated at 4°C for 30 minutes, and finally washed with 2 mL of FC buffer. Data were acquired using the BD FACSCelesta cytometer (BD Biosciences). Surface IgM MFI values were determined by subtracting the MFI of the isotype control, which was run in parallel for each sample. All FC data were analysed using FlowJo version 10.8 (TreeStar, Ashland, OR).

Quantitative real-time PCR (qPCR)

Total RNA was extracted using the RNAse Plus Mini Kit (Qiagen), retrotranscribed to complementary DNA using the High-Capacity cDNA Reverse Transcription Kit (ThermoFisher), and analyzed using the CFX384 Touch Real-time detection system (Bio-Rad, Hercules, CA). TaqMan Gene Expression Assays (Thermo Fisher Scientific) used are: Hs00984230_m1 (*B2M*); Hs00234142_m1 (*CCL3*); Hs00153408_m1 (*MYC*); Hs00971639_m1 (*COX4I1*); Hs02786624_g1 (*GAPDH*); Hs00273372_s1 (*TFAM*).

TFAM down-regulation

Down-regulation experiments were performed using Silencer Select siRNAs for controls and TFAM according to the manufacturer's specifications (Thermo Fisher, Silencer Select, Control, 4390843, TFAM, 4392420 – s14000). Cells were assessed at 48h after silencing to identify changes in TFAM protein levels and gene expression

MitoTracker Staining

FC analysis for mitochondrial mass evaluation was carried out as follows: 2×10^5 cells were washed with PBS and incubated for 30 min at room temperature with 10 nM MitoTracker Green (Thermo Fisher). After incubation, fluorescence was measured by BD FACScelesta.

Confocal microscopy for mitochondrial mass evaluation was carried out as follows: the fluorescent dye MitoTracker Deep Red FM (Thermo Fisher) was used to monitor mitochondrial morphology in living cells according to the manufacturer's instructions. Cells were imaged with a Leica Stellaris 5 confocal microscope (Leica Microsystems, Wetzlar, Germany) equipped with 4 excitation lasers (405 Diode, Argon, DPSS561, HeNe633). Images were acquired with a HCX PL APO 63×/1.4 NA oil-immersion objective on the three coordinates of the space (XYZ planes). High-resolution insets were captured at 40 nm (XY) and 300 nm (Z), while full-field acquisitions were acquired at 180 nm (XY) and 300 nm (Z). Image quality was enhanced using the LIGHTNING adaptive deconvolution module (Leica

Microsystems), which allows real-time resolution improvement by computationally reducing out-of-focus signal. Images were processed and analyzed with ImageJ software (Rasband, W.S., U.S. National Institutes of Health, Bethesda, MD). Quantitative analysis of mitochondrial surface area and volume was performed using the IMARIS software (Bitplane, Zurich, Switzerland).

Seahorse metabolic rate assay

Seahorse assay was performed for measuring Oxygen Consumption Rates (OCR) and Extracellular Acidification Rates (ECAR) in primary CLL cells. Specifically, OCR and ECAR were respectively measured with the XFp Mito Stress Test Kit and the XFp Glycolysis Stress Test Kit, using a Seahorse XFe24 Analyzer, with reagents and equipment supplied by Agilent Technologies. On the day before the assay, 5×10^{6} cells/well were seeded in a complete medium in a 24-well plate coated with α-IgM and incubated in 5% CO_2 at 37°C for 24 hours. One hour prior to the analysis, the growth medium was replaced with Seahorse XF Base Medium (DMEM without phenol red and sodium bicarbonate, supplemented with 1 mM pyruvate, 2 mM L-glutamine, and 10 mM glucose for the Mito Stress Test, or with 2mM L-glutamine for the Glycolysis Stress Test, pH 7.4). This step allows cells to adapt to a non-buffered and defined metabolic environment that does not interfere with the extracellular flux measurements. For the Mito Stress Test, the medium provides substrates for oxidative phosphorylation, while for the Glycolysis Stress Test glucose is omitted initially to monitor glycolytic activation upon its addition. 1×10^{6} cells/well were plated in poly-L-lysine-coated XF24 cell culture microplates and incubated 45 minutes at 37°C without CO_2 supplementation. The poly-L-lysine coating promotes cell adhesion to the plate bottom, which is required for accurate measurements by the Seahorse analyzer. The CO_2-free incubation ensures pH stability of the assay medium, which lacks bicarbonate buffering. For

the Mito Stress Test, during the assay, 1,5 µM oligomycin, 1 µM FCCP, and 0,5 µM rotenone/antimycin A were sequentially injected into each well in accordance with standard protocols. Oligomycin is an ATP synthase inhibitor that blocks mitochondrial ATP production, allowing calculation of ATP-linked respiration. FCCP is a protonophore that uncouples oxidative phosphorylation, enabling the assessment of maximal respiratory capacity. Rotenone and antimycin A inhibit complexes I and III of the electron transport chain, respectively, thereby halting mitochondrial respiration and allowing estimation of non-mitochondrial oxygen consumption For the Glycolysis Stress Test, cells were sequentially treated with 10 mM glucose, 1 µM oligomycin, and 50 mM 2-deoxyglucose, following the manufacturer's protocol. Glucose addition initiates glycolysis, leading to extracellular acidification. Oligomycin forces cells to rely solely on glycolysis by inhibiting mitochondrial ATP production. 2-deoxyglucose is a glucose analog that inhibits glycolysis by competitively blocking hexokinase, confirming the glycolytic nature of the measured acidification. Each patient measurement was performed in technical triplicates or quadruplicates on the same plate, and at least 6 independent patient samples were measured per genotype. The data were analyzed using Wave Seahorse software.

Cell homogenate preparation for enzymatic assays

Cells were centrifuged at 1000 rpm for 5 min, and the growth medium was removed. The pellet was washed in PBS twice and centrifuged again. The pellet was resuspended in PBS plus protease inhibitor and sonicated twice for 10 sec, in ice and with a 30 sec break to prevent the mixture from warming, using the Microson XL Model DU-2000 (Misonix Inc, Durham, NC). Total protein content was estimated with the Bradford method.

TCA cycle enzyme assay

For each assay 50 µg of total protein were used. Citrate synthase was assayed spectrophotometrically at 232 nm following the decomposition of acetyl-CoA. The assay mix contained: Tris-HCl (pH 8), 0.17 mM oxaloacetic acid, and 0.20 mM acetyl-CoA. 7 α-ketoglutarate dehydrogenase was assayed spectrophotometrically at 340 nm following NAD^+ reduction. The assay medium contained: Tris-HCl (pH 7.5), 5 mM $MgCl_2$, 40 mM rotenone, 2.5 M α-ketoglutarate, 0.1 mM CoA, 0.2 mM thiamine pyrophosphate, and 1 mM NAD^+.

Glucose consumption and lactate release

Glucose consumption in the growth medium was assessed by measuring NADP reduction at 340 nm. For this, 10 µl of the growth medium was added to a solution containing 50 mM Tris-HCl pH 8.0, 1 mM NADP, 10 mM $MgCl_2$, and 2 mM ATP. Spectrophotometric analysis of the samples was conducted before and after the addition of 4 µg of purified hexokinase (HK) and glucose-6-phosphate dehydrogenase (G6PD).

Lactate concentration in the growth medium was measured spectrophotometrically by monitoring NAD^+ reduction at 340 nm. The assay medium consisted of 10 µl of growth medium, 100 mM Tris-HCl pH 8.0-, and 5-mM NAD^+. Samples were analyzed spectrophotometrically before and after the addition of 4 µg of purified LDH. Both sets of data were normalized to the cell number.

ATP synthesis and Oxygen consumption rate (OCR)

Mitochondrial ATP synthesis was assayed in $2x10^5$ cells resuspended in a medium containing 10 mM Tris-HCl (pH 7.4), 50 mM KCl, 1 mM EGTA, 2 mM EDTA, 5 mM KH_2PO_4, 2 mM MgCl2, 0.6 mM ouabain, and 0.040 mg/ml ampicillin. The reaction was monitored using the luciferin/luciferase method (luciferin/luciferase ATP bioluminescence assay kit CLSII, Roche, Basel, Switzerland) at 25°C following the addition of respiratory substrates (i.e., 10 mM

pyruvate plus 5 mM malate or 20 mM to stimulate the pathways composed of complexes I, III, and IV, or II, III, and IV, respectively). The reaction started with the addition of 0.1 mM ADP and was monitored by the GloMax® 20/20 Luminometer (Promega, Madison, WI) over two minutes, with measurements taken every 30 seconds.

Oxygen consumption rate (OCR) was measured with an amperometric electrode (Unisense Microrespiration, Unisense A/S, Denmark) in a closed chamber at 25 °C. For each experiment, $2x10^5$ cells were resuspended in phosphate buffer saline (PBS) and permeabilized with 0.03 mg/ml digitonin for 1 min. As respiring substrates the same molecules used for ATP synthesis have been employed.

RNA sequencing experiment

RNA quality was assessed using the Bioanalyzer RNA 6000 Nano kit (Agilent Technologies), and RNA yield was quantified using the Qubit RNA HS assay kit (Life Technologies). Libraries were prepared using the Stranded Total RNA Prep Ligation with Ribo-Zero Plus (Illumina), following the manufacturer’s protocol. RNA sequencing (RNA-seq) was performed with a sample size of n = 2 for both MEC-1/N^{M} and MEC-1/N^{WT} cell lines per condition; each replicate consisting of a pool of 3 independently extracted RNAs. Raw FASTQ files were processed using the Illumina BaseSpace RNA-Seq Alignment application (v2.0.2), which performs spliced alignment using STAR and transcript quantification using Salmon, and exports transcript/gene abundance estimates as TPM. Reads were aligned to the UCSC hg19 human reference genome. For PCA and downstream transcriptome-level analyses, we restricted to protein-coding genes and retained genes with TPM > 0 in at least one sample, resulting in 20,395 genes used as input for PCA and related analyses.

Principal component analysis (PCA)

PCA was conducted using the PCAtools package (v2.12.0). TPM data were used as inputs, for a total of 20395 protein-coding genes. A pathway-specific PCA of the NOTCH1 signaling pathway was performed by taking only the genes annotated from PathCards (https://pathcards.genecards.org, NOTCH1 signaling pathway)

Gene Set Enrichment Analysis (GSEA)

GSEA was performed by using the GSEA software v4.3.2. A standard number of 1000 permutations was selected and “Ratio of Classes” was used as a ranking metric. The chip utilized was the Human Illumina array (Human_Illumina_HumanHT_12_v3_Array_MSigDB.v7.2.chip), whereas the MSigDB_Hallmark (v.2023.2) dataset was used for the analyses. “Permute” parameter was set on “gene_set”.

Heatmap

Heatmaps were generated through the ComplexHeatmap R package (v2.18.0) by plotting Z-score-normalized TPMs. Either metabolic-related genes (https://www.gsea-msigdb.org, Metabolism) or proliferative genes (https://www.gsea-msigdb.org, CELL_PROLIFERATION_GO_0008283) were used as inputs. K-means was used as clustering distance for the columns (k = 2). Each column consists of the average TPM values of the replicates of each clone (n = 2).

TFAM barplot

Trimmed Mean of M-values (TMM) normalization was performed using edgeR (v3.14.0), and expression levels of TFAM were visualized as a barplot using ggplot2 (v3.5.2)

Metabolic reaction enrichment analysis (MaREA)

MaREA was performed by using MaREA4Galaxy (http://marea4galaxy.cloud.ba.infn.it/galaxy/). Metabolic reactions with a p-value <0.05 and a fold change > |1.2| were selected as statistically significant.

RNA-seq data analysis on primary CLL cells

Gene-level TPM expression matrices for primary CLL samples were retrieved from the comprehensive study by Knisbacher et al.[3] via the CLL-map portal (www.cllmap.org). Only treatment-naïve cases with unmutated IGHV (IGHV-UM) were included for downstream analyses. Samples were stratified by *NOTCH1* mutational status (CLL/N^{WT} vs CLL/N^{M}). Because the number of eligible cases in the two classes was not identical, we applied a random down-sampling (class-balancing) procedure to obtain 50 CLL/N^{WT} and 50 CLL/N^{M} samples. A summary of the selected CLLmap cohort used for transcriptomic analyses is provided in Table S3A. GSEA analysis was performed likewise to MEC-1 clones, except for the ranking metric (Signal2Noise) and the “permute” parameter (set on “phenotype”).

Differential expression analysis on primary CLL samples was performed using DESeq2 (v1.48.2) on gene-level raw counts from the CLL-map portal. To account for potential cohort/batch effects in this multi-cohort dataset, we included a batch covariate derived from the cohort identifier encoded in participant_id (first character) in the DESeq2 design. Differential expression (*NOTCH1*-mutated vs *NOTCH1*-wild-type) was assessed using the DESeq2 Wald test, with Benjamini–Hochberg correction; significance was defined as FDR < 0.05 (and |log2FC| ≥ 1 where specified for downstream analyses). Volcano plots were generated from log2 fold changes and FDR-adjusted *P* values using ggplot2. For pathway-informed labeling, leading-edge (“core enrichment”) genes from GSEA (REACTOME_MITOCHONDRIAL_BIOGENESIS, HALLMARK_GLYCOLYSIS,

HALLMARK_OXIDATIVE_PHOSPHORYLATION) were intersected with DESeq2 results, filtered at FDR < 0.05, ranked by |log2FC|, and the top five genes per pathway were annotated together. GO analysis was performed for genes up- and down-regulated in CLL/N^{M} versus CLL/N^{WT} (FDR < 0.05 and |log2FC| ≥ 1), using the GO_Biological_Process_2023 database, and results were summarized by the top 20 enriched terms ranked by significance, using ggplot2.

CUT&Tag experiments

Briefly, cells were harvested, counted, and centrifuged at 1500 RPM for 5 min at room temperature. $10x10^6$ cells per condition were washed twice in wash buffer (20 mM HEPES-KOH pH 7.5, 150 mM NaCl, 0.5 mM Spermidine, 1x Protease inhibitor cocktail; Roche #11836170001). Nuclei were isolated by incubating cells for 10 minutes on ice in cold Nuclei Extraction buffer (NE1 buffer: 20 mM HEPES-KOH pH 7.9, 10 mM KCl, 0.1% Triton X-100, 20% Glycerol, 0.5 mM Spermidine, 1x Protease inhibitor cocktail). For NOTCH1 binding analysis, they were lightly cross-linked by adding 16% formaldehyde to a final concentration of 0.1%. Concanavalin A coated magnetic beads (Bangs Laboratories #BP531) were prepared and split into individual 0.2 ml tubes with isolated Nuclei (25.000 to analyze histone modifications, 50.000 to analyze NOTCH1 binding), then incubation with the primary antibody was performed at 4 °C overnight. All antibodies were from Cell Signaling (Danvers, MA) and were anti-NOTCH1 #3680 1:100 dilution; anti-H3K4me3 #9751 1:50 dilution; anti-H3K27me3 #9733 1:50 dilution; anti-H3K27ac #5326 1:100 dilution; anti-H3K4me1 #5326 1:50 dilution; anti-IgG control #2729 1:50 dilution. The next day, samples were washed with wash buffer and incubated at room temperature for 2 h with the secondary antibody (Guinea Pig Anti-Rabbit IgG #611-201-122, 1:200 dilution, Rockland Immunochemicals,

Rockland, MA). After, the samples were resuspended in 300-wash buffer (20 mM HEPES pH 7.5, 300 mM NaCl, 0.5 mM Spermidine, 1 × Protease inhibitor cocktail) containing pAG-Tn5 pre-loaded adapter complex (1:20 dilution) and incubated at RT for 1 h. Samples were washed twice with 300-wash buffer and tagmentation was performed with tagmentation buffer (300-wash buffer plus 10 mM MgCl2) for 1 h at 37 °C. To release Tn5 and prepare tagmented chromatin for PCR, samples were resuspended in SDS release solution (10mM TAPS pH 8.5, 0.1% SDS) for 1h at 58 °C. Neutralizing solution (0.67% Triton-X100) was added and PCR amplification was performed using NEBNext PCR mix and 2 µl of universal barcoded i5 primer (10 µM), 2 µl of barcoded i7 primer (10 µM)[4]. PCR amplification was performed using 14 rapid cycles following these conditions: 72°C for 5 min; 98°C for 30 s; 14 cycles of 98°C for 10 s and 63°C for 30 s; final extension at 72°C for 1 min; hold at 4°C. CUT&Tag libraries were cleaned with a single round of SPRI beads (Beckman Coulter, A63881, ratio of 1.8 ul of beads to 1L of PCR product), quantified on a TapeStation Bioanalyzer instrument, and pooled for sequencing on NextSeq 550 instrument (Illumina) using paired-end 75x75 readout with ~ 4 million reads per sample. FASTQ files were trimmed using Cutadapt vs 4.9 and aligned to the GRCh38 human reference genome using Bowtie2 vs 2.5.4. Peak calling was performed with MACS2 vs 2.1.0.

Differential binding affinity analysis was conducted using DiffBind vs 3.4.11 within the R environment 4.3.2, applying the edgeR method implemented in DiffBind.

Genomic regions exhibiting significant gains or losses in binding affinity were identified using a false discovery rate (FDR) < 0.05.

Peaks were annotated using the ChIPpeakAnno Bioconductor package vs 3.16.1, and genes associated with regions displaying differential binding affinity were used for downstream pathway enrichment analysis.

Peak calling shown in Figure 5A was performed using SEACR_1.3 in stringent mode, with IgG controls used to define background signal. Replicates (n = 3) for each sample were merged, and both bigWig signal tracks and BED peak files were visualized using IGV (v2.19.5).

Apoptosis Evaluation

MEC-1 clones were treated with V-9302, UK-5099, venetoclax, or their combinations (V-9302 + venetoclax, UK-5099 + venetoclax) for 72 or 96 hours. Primary CLL cells were treated with V-9302, venetoclax, or the combination of V-9302 + venetoclax for 24 or 48 hours. Additionally, MEC-1 clones transfected with TFAM-specific siRNA were assessed for apoptosis after 48 hours. Following treatment or siRNA transfection, cells were collected and assessed for viability using the Annexin V–APC Apoptosis Detection Kit (ThermoFisher), according to the manufacturer's instructions. Samples were analyzed by flow cytometry using a BD FACSCelesta. The synergistic effects on MEC-1 and CLL cells of the dual administration of Venetoclax/V9302 ($E_{Venetoclax/V9302}$) compared to the single agents ($E_{Venetoclax}$/E_{V9302}), at both doses used, in terms of apoptosis induction were evaluated by calculating the combination index (CI) as follow[6]: $CI=(E_{Venetoclax}+E_{V9302})-(E_{Venetoclax}E_{V9302})/E_{Venetoclax/V9302}$, with CI < 1, = 1, and > 1 indicating synergy, additivity, and antagonism, respectively.

Statistical analyses

Sample sizes were selected according to the expected biological variability, with fewer biological replicates for the relatively homogeneous MEC-1 cell line and more for the intrinsically heterogeneous primary CLL samples; the exact n for each experimental condition is reported in the figure legends. Data distribution and group variance were checked by plotting individual data points with summary statistics (mean ± SEM). Visual inspection did not show major deviations from approximate normality, similar variances or

independence of observations, indicating no relevant violations of the assumptions of the unpaired two-tailed t-tests; in all graphs, n refers to the number of biological replicates, center values are means, and error bars represent SEM, as specified in the figure legends.

REFERENCES

1. Arruga F, Bracciama V, Vitale N, et al. Bidirectional linkage between the B-cell receptor and NOTCH1 in chronic lymphocytic leukemia and in Richter's syndrome: therapeutic implications. *Leukemia*. 2020;34(2):462-477.
2. Arruga F, Gizdic B, Bologna C, et al. Mutations in NOTCH1 PEST domain orchestrate CCL19-driven homing of chronic lymphocytic leukemia cells by modulating the tumor suppressor gene DUSP22. *Leukemia*. 2017;31(9):1882-1893.
3. Knisbacher BA, Lin Z, Hahn CK, et al. Molecular map of chronic lymphocytic leukemia and its impact on outcome. *Nat Genet*. 2022;54(11):1664-1674.
4. Buenrostro JD, Wu B, Litzenburger UM, et al. Single-cell chromatin accessibility reveals principles of regulatory variation. *Nature*. 2015;523(7561):486-490.
5. Ye Zheng KA, Steven Henikoff. CUT&Tag Data Processing and Analysis Tutorial. *Springer Nature*.
6. Foucquier J, Guedj M. Analysis of drug combinations: current methodological landscape. *Pharmacol Res Perspect*. 2015;3(3):e00149.

TABLES

Table S1. Antibodies used in WB and FC.

**Western blot Primary antibody**	**Catalog number**	**Company**
anti-p-Akt	#9271	Cell Signaling Technologies
anti-Akt	#9272	Cell Signaling Technologies
anti-p-Btk	#5082S	Cell Signaling Technologies
anti-Btk	#8547	Cell Signaling Technologies
anti-p-Erk	#4377	Cell Signaling Technologies
anti-Erk	#610124	BD Transduction Laboratories
anti-p-PLCγ2	#3874	Cell Signaling Technologies
anti-PLCγ2	#3872	Cell Signaling Technologies
anti- Cleaved Notch1 (Val1744)	#4147	Cell Signaling Technologies
anti-β-actin HRP conjugated	#12620	Cell Signaling Technologies
**Western blot Secondary antibodies**	**Catalog number**	**Company**
anti-rabbit IgG HRP-conjugated	#GEHNA9341ML	Cytiva
anti-mouse IgG HRP-conjugated	#GEHNA9311ML	Cytiva

**Flow cytometry antibodies**	**Catalog number**	**Company**
anti-human CD19 PE-Cy7	#557835	BD Pharmingen
anti-human IgM PE-Cy7	#314532	BD Pharmingen

anti-human IgM PE	#555783	BD Pharmingen
anti-human CD5 BB515	#564647	BD Pharmingen
anti-human CD3 APC	#17003742	eBioscience
anti IgG2a κ, Rat, PE Cy7 Isotype Control	#552784	BD Pharmingen

Table S2A. Main clinical, genetic/cytogenetic, and molecular features of CLL patients included in the analyses. (WT = wild-type; mut = mutated; F = female; M = male; NA = not available; Del = deletion; tris = trisomy).

ID	*NOTCH1* mut	IGHV	tris12	del17	del11	del11/17	del13	p53 inact
P1	M	UM	0	0	0	0	1	
P2	M	UM	0	0	0	0	0	0
P3	WT	UM	1	0	0	0	0	0
P4	WT	UM	0	0	0	1	1	1
P5	WT	UM	0	1	1	1	1	1
P6	M	UM	0	0	0	0	1	0
P7	WT	UM	0	0	0	0	1	0
P8	WT	UM	1	0	1	1	1	0
P9	WT	UM	0	0	0	1	1	1
P10	WT	UM	0	0	0	0	1	0
P11	M	UM	0	0	0	0	1	0
P12	M	UM	1	0	0	0	0	nd
P13	WT	UM	0	0	0	1	0	0
P14	M	UM	nd	nd	nd	nd	nd	nd
P15	M	UM	0	0	0	0	0	nd
P16	M	UM	1	1	0	1	0	nd
P17	M	UM	1	0	0	0	0	nd
P18	WT	UM	0	0	1	1	1	nd
P19	WT	UM	0	0	1	1	0	nd
P20	M	UM	1	0	0	0	0	nd
P21	WT	UM	0	1	1	0	0	nd
P22	M	UM	0	0	0	0	0	0

P23	M	UM	1	0	0	0	0	0
P24	WT	UM	1	0	0	0	0	0
P25	M	UM	0	0	0	0	1	0
P26	WT	UM	1	0	0	0	0	nd
P27	WT	UM	0	0	1	1	1	0
P28	WT	UM	0	0	0	0	1	0
P29	M	UM	0	0	0	0	0	0
P30	WT	UM	0	0	0	0	0	0
P31	WT	UM	0	0	0	0	0	0
P32	M	UM	0	0	0	0	0	0
P33	M	UM	0	0	0	0	0	0
P34	M	UM	0	0	0	0	0	0
P35	WT	UM	0	0	0	0	1	1
P36	WT	UM	0	0	0	0	1	0
P37	M	UM	0	0	0	0	1	1
P38	M	UM	0	0	1	1	1	0
P39	WT	UM	0	0	0	0	0	0
P40	WT	UM	0	0	0	0	0	0
P41	M	UM	0	0	0	0	1	0
P42	WT	UM	0	0	1	1	0	0
P43	WT	UM	0	0	0	0	0	0
P44	M	UM	0	0	0	0	0	0
P45	M	UM	1	0	0	0	1	0
P46	WT	UM	0	0	0	0	1	0
P47	WT	UM	1	0	0	0	0	0
P48	WT	UM	1	0	0	0	0	0
P49	WT	UM	nd	nd	nd	nd	nd	nd

P50	M	UM	1	0	0	0	0	nd
P51	WT	UM	0	0	1	1	1	nd
P52	WT	UM	0	0	0	0	0	nd
P53	WT	UM	0	0	0	0	1	nd
P54	WT	UM	0	0	0	0	1	nd
P55	WT	UM	0	0	0	0	1	nd
P56	WT	UM	0	0	0	0	1	nd
P57	WT	UM	1	0	0	0	0	nd
P58	M	UM	1	0	0	0	0	nd
P59	M	UM	0	0	0	0	0	0
P60	WT	UM	0	0	1	0	0	0
P61	M	UM	nd	nd	nd	nd	nd	nd
P62	WT	UM	0	0	0	0	1	0
P63	M	UM	1	0	0	0	0	0
P64	M	UM	0	0	0	0	1	0

Table S2B. Statistical comparison of cytogenetic abnormalities between CLL/N^{WT} (n = 36) and CLL/N^{M} (n = 28). Values are n/N (%) among patients with available data for the given feature, where N is the sum of CLL/N^{WT} and CLL/N^{M}; missing values are reported and were excluded from hypothesis testing. Two-sided Fisher's exact test was used for 2×2 comparisons.

Variable	NWT (n=36)	NM (n=28)	p-value (Fisher)
IGHV unmutated (UM)	36/36 (100%)	28/28 (100%)	-
tris(12)	7/35 (20.0%), missing 1	9/26 (34.6%), missing 2	0.246

del(13q)	18/35 (51.4%), missing 1	9/26 (34.6%), missing 2	0.207
del(11q)	9/35 (25.7%), missing 1	1/26 (3.8%), missing 2	0.034
del(17p)	2/35 (5.7%), missing 1	1/26 (3.8%), missing 2	1.000
del(11q)+del(17p) (del11/17)	10/35 (28.6%), missing 1	2/26 (7.7%), missing 2	0.055
TP53 inactivation	4/24 (16.7%), missing 12	1/18 (5.6%), missing 10	0.371

Table S2C. Number and sex distribution of CLL samples analyzed in each figure, showing total cases, *NOTCH1* status (WT = wild-type; M = mutated), and sex (M = male; F = female).

**Figure/Experiment**	**n (total)**	**CLL/N^{WT} (n)**	**CLL/N^{M} (n)**	**M (n)**	**F (n)**	**M (%)**	**F (%)**
Figure 1A/ Western Blot	18	9	9	12	6	66,67	33,33
Figure 1B/ qtRT-PCR *DTX*	23	14	9	14	9	60,87	39,13
Figure 1B/ qtRT-PCR *NRARP*	16	9	7	9	7	56,25	43,75
Figure 2A/ Glycolysis Stress Test	19	11	7	15	4	78,95	21,05
Figure 2B/ Mitostress Test	19	11	8	14	5	73,68	26,32
Figure 2D/ qtRT-PCR *COX4I1/GAPDH*	16	7	9	11	5	68,75	31,25
Figure 5B/ qtRT-PCT *TFAM*	10	3	4	5	2	71,43	28,57
Figure 7E/ Cell viability 48h	12	5	8	9	3	75,00	25,00

Supplementary Fig.1A/ Glucose consumption	6	3	3	2	4	33,33	66,67
Supplementary Fig.1B/ Lactate concentration	6	3	3	2	4	33,33	66,67
Supplementary Fig.1C/ Proton Leak	19	11	8	14	5	73,68	26,32
Supplementary Fig.1D/ Spare Respiratory Capacity	19	11	8	14	5	73,68	26,32
Supplementary Fig.1E/ MitoSOX	7	3	4	4	3	57,14	42,86
Supplementary Fig.1E/ MitoTracker Green	12	7	5	8	4	66,67	33,33
Supplementary Figure 6/ Metabolomic isotopologue analysis	6	3	3	5	1	83,33	16,67
Supplementary Figure 7A/ Glutamine uptake	7	3	4	6	1	85,71	14,29
Supplementary Figure 7A/ Glucose uptake	17	7	10	11	6	64,71	35,29
Supplementary Figure 9B/ Cell viability 24h	12	5	8	9	3	75,00	25,00

Table S3. Summary table of N^{M} and N^{WT} CLL patients from the cohort of Knisbacher et al.

Characteristic	(*NOTCH1* mutation) no<br>N = 50	(*NOTCH1* mutation) yes<br>N = 50
participant_id		
CRC-0012	0 (0%)	1 (2.0%)

CRC-0015	0 (0%)	1 (2.0%)
DFCI-5018	0 (0%)	1 (2.0%)
DFCI-5051	0 (0%)	1 (2.0%)
DFCI-5066	1 (2.0%)	0 (0%)
DFCI-5068	1 (2.0%)	0 (0%)
DFCI-5142	1 (2.0%)	0 (0%)
DFCI-5156	0 (0%)	1 (2.0%)
DFCI-5165	0 (0%)	1 (2.0%)
DFCI-5172	0 (0%)	1 (2.0%)
DFCI-5174	0 (0%)	1 (2.0%)
DFCI-5195	1 (2.0%)	0 (0%)
GCLL-0006	1 (2.0%)	0 (0%)
GCLL-0009	1 (2.0%)	0 (0%)
GCLL-0028	0 (0%)	1 (2.0%)
GCLL-0029	1 (2.0%)	0 (0%)
GCLL-0035	0 (0%)	1 (2.0%)
GCLL-0043	1 (2.0%)	0 (0%)
GCLL-0045	0 (0%)	1 (2.0%)
GCLL-0048	0 (0%)	1 (2.0%)
GCLL-0050	1 (2.0%)	0 (0%)
GCLL-0055	1 (2.0%)	0 (0%)
GCLL-0066	0 (0%)	1 (2.0%)
GCLL-0091	0 (0%)	1 (2.0%)
GCLL-0092	0 (0%)	1 (2.0%)
GCLL-0099	1 (2.0%)	0 (0%)
GCLL-0108	1 (2.0%)	0 (0%)
GCLL-0112	1 (2.0%)	0 (0%)
GCLL-0119	0 (0%)	1 (2.0%)
GCLL-0128	0 (0%)	1 (2.0%)
GCLL-0130	0 (0%)	1 (2.0%)
GCLL-0135	1 (2.0%)	0 (0%)
GCLL-0144	0 (0%)	1 (2.0%)
GCLL-0145	0 (0%)	1 (2.0%)
GCLL-0157	1 (2.0%)	0 (0%)
GCLL-0158	1 (2.0%)	0 (0%)

GCLL-0163	1 (2.0%)	0 (0%)
GCLL-0168	1 (2.0%)	0 (0%)
GCLL-0170	0 (0%)	1 (2.0%)
GCLL-0173	1 (2.0%)	0 (0%)
GCLL-0181	1 (2.0%)	0 (0%)
GCLL-0187	1 (2.0%)	0 (0%)
GCLL-0191	0 (0%)	1 (2.0%)
GCLL-0192	0 (0%)	1 (2.0%)
GCLL-0256	1 (2.0%)	0 (0%)
GCLL-0307	1 (2.0%)	0 (0%)
GCLL-0309	1 (2.0%)	0 (0%)
GCLL-0310	0 (0%)	1 (2.0%)
JB-0006	0 (0%)	1 (2.0%)
JB-0011	0 (0%)	1 (2.0%)
JB-0012	0 (0%)	1 (2.0%)
JB-0031	1 (2.0%)	0 (0%)
NHLBI-0034	1 (2.0%)	0 (0%)
NHLBI-0045	1 (2.0%)	0 (0%)
SCLL-0005	1 (2.0%)	0 (0%)
SCLL-0012	0 (0%)	1 (2.0%)
SCLL-0014	0 (0%)	1 (2.0%)
SCLL-0015	0 (0%)	1 (2.0%)
SCLL-0023	1 (2.0%)	0 (0%)
SCLL-0034	1 (2.0%)	0 (0%)
SCLL-0037	0 (0%)	1 (2.0%)
SCLL-0038	1 (2.0%)	0 (0%)
SCLL-0044	1 (2.0%)	0 (0%)
SCLL-0051	0 (0%)	1 (2.0%)
SCLL-0056	1 (2.0%)	0 (0%)
SCLL-0064	0 (0%)	1 (2.0%)
SCLL-0068	1 (2.0%)	0 (0%)
SCLL-0076	1 (2.0%)	0 (0%)
SCLL-0091	0 (0%)	1 (2.0%)
SCLL-0139	1 (2.0%)	0 (0%)
SCLL-0163	1 (2.0%)	0 (0%)

SCLL-0174	0 (0%)	1 (2.0%)
SCLL-0177	1 (2.0%)	0 (0%)
SCLL-0179	0 (0%)	1 (2.0%)
SCLL-0191	0 (0%)	1 (2.0%)
SCLL-0201	0 (0%)	1 (2.0%)
SCLL-0227	0 (0%)	1 (2.0%)
SCLL-0254	0 (0%)	1 (2.0%)
SCLL-0259	1 (2.0%)	0 (0%)
SCLL-0260	0 (0%)	1 (2.0%)
SCLL-0287	1 (2.0%)	0 (0%)
SCLL-0296	0 (0%)	1 (2.0%)
SCLL-0310	0 (0%)	1 (2.0%)
SCLL-0334	1 (2.0%)	0 (0%)
SCLL-0335	1 (2.0%)	0 (0%)
SCLL-0352	0 (0%)	1 (2.0%)
SCLL-0361	1 (2.0%)	0 (0%)
SCLL-0389	1 (2.0%)	0 (0%)
SCLL-0400	0 (0%)	1 (2.0%)
SCLL-0417	1 (2.0%)	0 (0%)
SCLL-0422	0 (0%)	1 (2.0%)
SCLL-0425	0 (0%)	1 (2.0%)
SCLL-0431	1 (2.0%)	0 (0%)
SCLL-0439	1 (2.0%)	0 (0%)
SCLL-0446	1 (2.0%)	0 (0%)
SCLL-0452	0 (0%)	1 (2.0%)
SCLL-0464	1 (2.0%)	0 (0%)
SCLL-0485	0 (0%)	1 (2.0%)
SCLL-0486	1 (2.0%)	0 (0%)
SCLL-0497	0 (0%)	1 (2.0%)
**treatment_status_at_samp**		
Untreated	50 (100%)	50 (100%)
**prior_treatment_category**		
Untreated	50 (100%)	50 (100%)
**IGHV_mut**		
unmutated	50 (100%)	50 (100%)

**Notch_mut_type**		
frameshift	0 (0%)	42 (84%)
missense	0 (0%)	3 (6.0%)
nonsense	0 (0%)	5 (10%)
WT	50 (100%)	0 (0%)

Table S4. Gene sets enriched in N^M CLL patients (n = 50) from the MsigDB_Hallmark_2020 dataset

	MsigDB_Hallmark_2020	SIZE	ES	NES	NOM p-val	FDR q-val	FWER p-val	RANK AT MAX
1	HALLMARK_PI3K_AKT_MTOR_SIGNALING	104	0.59	1.76	0.002	0.111	0.048	10745
2	HALLMARK_INTERFERON_GAMMA_RESPONSE	197	0.56	1.65	0.006	0.184	0.120	12215
3	HALLMARK_MITOTIC_SPINDLE	197	0.59	1.64	0.008	0.128	0.123	12581
4	HALLMARK_P53_PATHWAY	192	0.48	1.62	0.014	0.120	0.144	11787
5	HALLMARK_REACTIVE_OXYGEN_SPECIES_PATHWAY	47	0.57	1.61	0.031	0.109	0.161	10620
6	HALLMARK_UV_RESPONSE_UP	153	0.50	1.61	0.020	0.092	0.163	14377
7	HALLMARK_PROTEIN_SECRETION	95	0.57	1.59	0.039	0.094	0.181	13117
8	HALLMARK_INTERFERON_ALPHA_RESPONSE	95	0.59	1.55	0.045	0.110	0.222	12482
9	HALLMARK_APICAL_JUNCTION	191	0.49	1.53	0.028	0.116	0.251	16019
10	HALLMARK_WNT_BETA_CATENIN_SIGNALING	42	0.55	1.52	0.029	0.116	0.270	12639
11	HALLMARK_NOTCH_SIGNALING	32	0.55	1.52	0.046	0.106	0.270	15015
12	HALLMARK_HEME_METABOLISM	192	0.48	1.51	0.038	0.104	0.279	12186
13	HALLMARK_TGF_BETA_SIGNALING	54	0.53	1.51	0.038	0.097	0.281	16753
14	HALLMARK_APOPTOSIS	159	0.47	1.50	0.036	0.100	0.297	17613
15	HALLMARK_IL2_STAT5_SIGNALING	194	0.47	1.50	0.042	0.094	0.300	14362
16	HALLMARK_UNFOLDED_PROTEIN_RESPONSE	106	0.49	1.47	0.081	0.104	0.329	15014
17	HALLMARK_COMPLEMENT	199	0.46	1.45	0.067	0.112	0.354	17667
18	HALLMARK_MTORC1_SIGNALING	194	0.48	1.45	0.092	0.111	0.366	16500
19	HALLMARK_GLYCOLYSIS	197	0.45	1.42	0.082	0.123	0.408	14877
20	HALLMARK_ALLOGRAFT_REJECTION	195	0.44	1.41	0.071	0.126	0.416	15178
21	HALLMARK_CHOLESTEROL_HOMEOSTASIS	73	0.45	1.41	0.062	0.121	0.416	20201
22	HALLMARK_APICAL_SURFACE	43	0.48	1.38	0.101	0.138	0.453	13902
23	HALLMARK_ESTROGEN_RESPONSE_EARLY	193	0.41	1.38	0.076	0.134	0.454	11970
24	HALLMARK_HEDGEHOG_SIGNALING	35	0.49	1.36	0.067	0.141	0.472	4219
25	HALLMARK_ADIPOGENESIS	192	0.42	1.35	0.115	0.147	0.489	17439

26	HALLMARK_PEROXISOME	104	0.44	1.35	0.137	0.143	0.490	14129
27	HALLMARK_INFLAMMATORY_RESPONSE	197	0.45	1.34	0.121	0.144	0.500	13902
28	HALLMARK_FATTY_ACID_METABOLISM	155	0.44	1.33	0.168	0.142	0.503	21684
29	HALLMARK_DNA_REPAIR	146	0.44	1.33	0.170	0.139	0.507	17639
30	HALLMARK_OXIDATIVE_PHOSPHORYLATION	184	0.45	1.32	0.186	0.144	0.525	17439
31	HALLMARK_MYOGENESIS	197	0.42	1.31	0.109	0.143	0.531	14934
32	HALLMARK_ANDROGEN_RESPONSE	96	0.42	1.29	0.150	0.156	0.556	16658
33	HALLMARK_IL6_JAK_STAT3_SIGNALING	87	0.44	1.29	0.154	0.154	0.558	12482
34	HALLMARK_G2M_CHECKPOINT	190	0.44	1.29	0.199	0.151	0.558	12994
35	HALLMARK_HYPOXIA	191	0.37	1.27	0.130	0.157	0.572	16269
36	HALLMARK_UV_RESPONSE_DN	138	0.41	1.27	0.162	0.156	0.577	17525
37	HALLMARK_XENOBIOTIC_METABOLISM	196	0.39	1.26	0.180	0.157	0.587	14853
38	HALLMARK_KRAS_SIGNALING_DN	194	0.36	1.24	0.142	0.166	0.616	14630
39	HALLMARK_SPERMATOGENESIS	133	0.37	1.24	0.172	0.166	0.621	16050
40	HALLMARK_MYC_TARGETS_V2	57	0.44	1.21	0.277	0.188	0.663	20836
41	HALLMARK_BILE_ACID_METABOLISM	112	0.36	1.14	0.286	0.238	0.722	9554
42	HALLMARK_COAGULATION	137	0.35	1.11	0.305	0.264	0.756	15550
43	HALLMARK_KRAS_SIGNALING_UP	193	0.33	1.09	0.322	0.283	0.779	14066
44	HALLMARK_ESTROGEN_RESPONSE_LATE	194	0.31	1.06	0.365	0.303	0.800	14027
45	HALLMARK_E2F_TARGETS	195	0.36	1.04	0.419	0.312	0.804	12888
46	HALLMARK_TNFA_SIGNALING_VIA_NFKB	197	0.40	1.02	0.451	0.327	0.817	14650
47	HALLMARK_PANCREAS_BETA_CELLS	40	0.30	0.92	0.562	0.448	0.875	9394
48	HALLMARK_EPITHELIAL_MESENCHYMAL_TRANSITION	195	0.30	0.90	0.578	0.467	0.888	9404
49	HALLMARK_MYC_TARGETS_V1	193	0.31	0.89	0.599	0.466	0.891	16338

Table S5. Gene sets enriched in CLL/N^{M} patients (n = 50) from the Reactome_Mitochondrial_Biogenesis dataset

	Reactome_Mitochondrial_Biogenesis	SIZE	ES	NES	NOM p-val	FDR q-val	FWER p-val	RANK AT MAX
1	REACTOME_MITOCHONDRIAL_BIOGENESIS	73	0.62	1.90	0.000	0.000	0.000	12666

Table S6. Gene Ontology (GO Biological Process) terms related to mitochondrial function identified in the CUT&Tag differential enrichment analysis (H3K4me3), comparing MEC-1/N^{M} vs MEC-1/N^{WT} under basal conditions, highlighting upregulated pathways in MEC-1/N^{M} cells. Term: GO/pathway name returned by the enrichment. Overlap: shown as k/K, where k = NM-enriched genes (H3K4me3 higher in MEC-1/N^{M} vs MEC-1/N^{WT}) that fall in this term; K = total genes in that GO term. P.value: enrichment *P* value from the over-representation test. Adjusted.P.value: *P* value after Benjamini-Hochberg correction. Odds.Ratio: strength of enrichment (>1 = over-represented in MEC-1/N^{M}). Combined.Score: tool-specific score combining significance and effect size, used to rank terms. Genes: MEC-1/N^{M}-enriched genes contributing to this term.

Term	Overlap (k/K)	P.value	Adjusted.P.value	Odds.Ratio	Combined.Score	Genes
Mitochondrial RNA 3'-End Processing (GO:0000965)	4/5	0.00188129	0.41521568	23.8773092	149.84911	SUPV3L1;TRMT10C;TRNT1;HSD17B10
Ribosome Disassembly (GO:0032790)	5/12	0.02004083	0.76572533	4.26379757	16.6713783	MCTS1;HBS1L;TRIP4;MTIF3;MRRF
Mitochondrial Translation (GO:0032543)	22/98	0.02036231	0.76572533	1.73125773	6.74163798	GADD45GIP1;NDUFA7;MRPS27;MRPS14;MRPL18;MRPS31;MRPL17;MRPL28;MRPS10;GATB;MRPL48;MTIF3;MRPL35;MRPL46;MRPL54;MRPL22;MRPL44;MRPL30;MRPL52;LARS2;MTRF1;MTG1

Table S7. Gene Ontology (GO Biological Process) terms related to mitochondrial function identified in the CUT&Tag differential enrichment analysis (H3K4me3), comparing MEC-1/N^{M} vs MEC-1/N^{WT} under α-IgM stimulation, highlighting pathways upregulated in MEC-1/N^{M} cells. Term: GO/pathway name returned by the enrichment. Overlap: shown as k/K, where k = N^{M}-enriched genes (H3K4me3 higher in MEC-1/N^{M} vs MEC-1/N^{WT}) that fall in this term; K = total genes in that GO term. P.value: enrichment *P* value from the over-representation test. Adjusted.P.value: *P* value after Benjamini-Hochberg correction. Odds.Ratio: strength of enrichment (>1 = over-represented in MEC-1/N^{M}). Combined.Score: tool-specific score combining significance and effect size, used to rank terms. Genes: MEC-1/N^{M}-enriched genes contributing to this term.

**Term**	**Overlap (k/K)**	**P.value**	**Adjusted.P.value**	**Odds.Ratio**	**Combined.Score**	**Genes**
Mitochondrial Genome Maintenance (GO:0000002)	4/11	0.03011919	0.70628026	4.37756871	15.3328407	PARP1;LONP1;SLC25A36;TYMP
Regulation Of Establishment Of Protein Localization To Mitochondrion (GO:1903747)	3/7	0.03772723	0.72532059	5.74404504	18.8253795	DNAJA1;SREBF1;PARL

Supplementary Figure legends

Supplementary Fig. 1 (A) Pathway-level summary plot of Gene Set Enrichment Analysis (GSEA) results for the MSigDB Hallmark collection in primary CLL samples (CLL/N^{M} vs CLL/N^{WT}). Each point represents one Hallmark gene set, plotted by normalized enrichment score (NES) versus -log10(FDR q-value). Pathways discussed in the manuscript, including NOTCH signaling, glycolysis, oxidative phosphorylation, reactive oxygen species, and PI3K-AKT-mTOR signaling, are highlighted. The dashed line indicates the conventional GSEA significance threshold (FDR = 0.25).

(B) Gene Ontology (GO) Biological Process enrichment analysis of genes up-regulated (top) and down-regulated (bottom) in CLL/N^{M} versus CLL/N^{WT} samples, based on DESeq2 differential expression results. Bars represent -log10(p-value) for significantly enriched GO terms. Up-regulated genes show enrichment for mitochondrial respiratory, energy metabolism, and translational processes, whereas down-regulated genes are enriched for immune-related signaling pathways.

(C) Volcano plot showing differential gene expression between CLL/N^{M} and CLL/N^{WT} samples. Log2 fold change is plotted against -log10(FDR), with genes colored by direction of change (up-regulated, down-regulated, not significant). Labeled genes correspond to a representative subset of pathway-relevant genes selected from GSEA leading-edge (“core enrichment”) subsets of REACTOME_MITOCHONDRIAL_BIOGENESIS, HALLMARK_GLYCOLYSIS, and HALLMARK_OXIDATIVE_PHOSPHORYLATION as described in the Supplementary Methods section.

Supplementary Fig. 2. CLL/N^{M} displayed higher glycolytic and mitochondrial features than CLL/N^{WT}. (A) Glucose consumption by CLL/N^{WT} (n = 3) and CLL/N^{M} cells (n = 3).(B) Lactate concentration in the culture medium of CLL/N^{WT} (n = 3) and CLL/N^{M} cells (n = 3). (C) Proton

leak calculated from minimum measurement after oligomycin minus non-mitochondrial respiration (CLL/N^{WT} n=11, CLL/N^{M} n=8). (D) Spare respiratory capacity calculated from maximum measurement after DNP minus last measurement before oligomycin for CLL/N^{WT} and CLL/N^{M} cells either unstimulated (-) or stimulated (+) with plate-bound α-IgM for 24 hours (CLL/N^{WT} n=11, CLL/N^{M} n = 8). (E) (left) Representative flow cytometry profile of MitoSOX Red internalized by untreated (-) or stimulated (+) CLL/N^{WT} and CLL/N^{M} cells compared to unstained control. (E) (right) MFI of MitoSOX Red internalized by CLL/N^{WT} and CLL/N^{M} cells either unstimulated (-) or stimulated (+) with plate-bound α-IgM for 24 hours (CLL/N^{WT} n = 3, CLL/N^{M} n = 4). (F) Representative flow cytometry profile of MitoTracker Green chosen from two different CLL patients (CLL/N^{WT-}pink and CLL/N^{M}-blue, left panel). (right) MFI of MitoTracker Green internalized by unstimulated CLL/N^{WT} (n = 7) and CLL/N^{M} (n = 5). ($p \le 0.05$, *; $p \le 0.01$, **; $p \le 0.001$, ***; $p \le 0.0001$, ****)

Supplementary Fig. 3. Generation and validation of cell line models carrying an unmutated BCR with or without *NOTCH1* mutations.

(**A**) Graphical representation of the heterogeneous surface expression of IgM in the CLL-like cell line MEC-1. While most of the cells express sIgM (MEC-1^{IgM-M}N^{WT}, in yellow), a subpopulation is negative (MEC-1/sIgM^{-}N^{WT}, in pink). (**B**) Flow cytometry profile of sIgM expression in MEC-1 cells compared to isotype control (grey). (**C**) MEC-1/sIgM^{-} cells, which carry a *NOTCH1*-wild-type (N^{WT}) background, were genome-edited to introduce the *NOTCH1* mutation, generating MEC-1/sIgM^{-}/N^{M} clones. Both MEC-1/N^{WT} and MEC-1/N^{M} cells were then reconstituted with an unmutated BCR cloned from a CLL patient (CLL#1 – Table S2) carrying IGHV 4-59 genes, as MEC-1 cells. (**D**) Flow cytometry profile for surface IgM expression of the different MEC-1 clones. Isotype control is shown in grey.

Supplementary Fig. 4. Short-term BCR stimulation triggers a robust signalling cascade both in MEC-1/N^{WT} and in MEC-1/N^{M} cells

(**A**) Representative Western Blot of MEC-1/N^{WT} and MEC-1/N^{M} cell lines either not treated or stimulated for 1, 5 or 10 minutes with soluble α-IgM. Actin was used as loading control. (**B**) Quantitative optical density analysis of the ratio of the immunoreactive bands p-BTK/BTK, p-ERK/ERK, p-AKT/AKT and p-PLCγ2/PLCγ2. Band intensities were measured using Bio-Rad Image Lab and are shown as fold change normalized over the untreated (UN) bands. Asterisks refer to the significance compared to the not NT term. (**C**) qRT-PCR analysis of *CCL3* and *MYC* expression in MEC-1/N^{WT} and MEC-1/N^{M} cell lines either unstimulated (-) or stimulated (+) with plate-bound α-IgM for 6 hours. mRNA expression was normalized over β*2 microglobulin* (*β2M*) (n > 3). (**D**) qRT-PCR analysis of *NRAP* expression in MEC-1/N^{WT} (n = 6) and MEC-1/N^{M} (n = 5) cells following stimulation with anti-IgM and/or EDTA, as indicated. mRNA expression was normalized over β-2-microglobulin (*β2M*). ($p \leq 0.05$, *; $p \leq 0.01$, **; $p \leq 0.001$, ***; $p \leq 0.0001$, ****).

Supplementary Fig. 5 Pairwise principal component analysis (PCA) projections of the MEC-1 RNA-seq cohort (n = 100; 50 MEC-1/N^{WT} and 50 MEC-1/N^{M} samples). All combinations of the first five principal components (PC1-PC5) are shown. Points are colored by stimulation condition (IgM vs NT) and shaped by *NOTCH1* mutational status (N^{M} vs N^{WT}), as indicated in the legend. The percentage of variance explained by each principal component is reported on the corresponding axis.

Supplementary Fig. 6. Analysis of the transcriptomic landscape of MEC-1/N^{WT} and MEC-1/N^{M} cells.

(**A**) Principal component analysis (PCA) of MEC-1/N^{WT} and MEC-1/N^{M} cell line either unstimulated (-) or stimulated (+) with plate-bound α-IgM for 24 hours. TPM data were used

as inputs, for a total of 20,395 protein coding genes. Circles indicate stimulated cells whereas triangles unstimulated ones. Samples colored in pink indicate MEC-1/N^{M} cells, while samples colored in blue indicate MEC-1/N^{M} ones. (**B**) PCA of MEC-1/N^{WT} and MEC-1/N^{M} cell line either unstimulated or stimulated. TPM data were used as inputs, for a total of 100 genes belonging to the NOTCH1 signaling pathway. PCA loadings, meaning the genes better explaining the separation between samples, are also shown. (**C**) Gene Set Enrichment Analyses (GSEA). Cumulative graph of the main gene sets modulated in unstimulated (UN) MEC-1/N^{M} vs. MEC-1/N^{WT} cells (left panel). Cumulative graph of the main gene sets modulated in MEC-1/N^{M} vs. MEC-1/N^{WT} cells stimulated (IgM) with plate-bound α-IgM for 24 hours (right panel). Normalized enrichment score (NES) for each significant gene set (p-value < 0.05) is plotted. (**D**) Heatmap showing the gene expression profile in MEC-1/N^{WT} vs. MEC-1/N^{M} cells (n = 2), in both basal and stimulated conditions. Vertical dendrograms show correlation between samples. Red and blue colors represent higher and lower transcriptionally expressed genes, respectively, in the samples. Z-scores-transformed TPMs were plotted. Each column consists of the average TPM values of the replicates of each clone. Heatmap on the left was made with metabolic-related genes only (>2000 genes), whereas the one in the right with genes implicated in proliferation.

Supplementary Fig. 7. HMRcore map enriched by MaREA of MEC-1/N^{M} and MEC-1/N^{WT} cell line models in basal conditions. Reactions up-regulated in MEC-1/N^{M} are marked in red, and reactions up-regulated in MEC-1/N^{WT} are marked in blue. Thickness of the edges is proportional to the fold-change. Dashed gray arrows refer to non-significant deregulations (p-value > 0.05). Non-classified reactions, meaning reactions without information about the corresponding gene-enzyme rule, are marked in black. Dashed boxes highlight key

pathways, including the pentose phosphate pathway (PPP), oxidative phosphorylation, fatty acid synthesis, glutamine metabolism and nucleotide synthesis.

Supplementary Fig. 8. Glucose and glutamine fueling of BCR-stimulated CLL/N^{WT} and CLL/N^{M} cells.

(**A**) Metabolomic isotopologue analysis was performed in CLL/N^{WT} and CLL/N^{M} cells stimulated with plate-bound α-IgM for 24 hours and subsequently cultured in medium containing either [U-^{13}C]-glucose (orange) and [U-^{13}C]-glutamine (green) (n = 3). Incorporation of ^{13}C in metabolic intermediates was analyzed by liquid chromatography–mass spectrometry (LC-MS). (**B**) Mass isotopologue distributions (MID) of each m+n isotopologue were plotted to assess ^{13}C incorporation from fully labelled glucose (CLL/N^{WT}, yellow; CLL/N^{M}, orange) or glutamine (CLL/N^{WT}, light blue; CLL/N^{M}, dark blue) into selected metabolites following BCR stimulation. Data represent mean ± SD. ($p \leq 0.05$, *; $p \leq 0.01$, **; $p \leq 0.001$, ***; $p \leq 0.0001$, ****).

Supplementary Fig. 9. Glutamine and glucose uptake in both cell lines and CLL

(**A**) Bar plot showing the glutamine uptake of CLL/N^{WT} (n = 3) and CLL/N^{M} (n = 4) (left) and of MEC-1/N^{WT} (n = 5) and MEC-1/N^{M} cells (n = 4)(right) either unstimulated (-) or stimulated (+) with plate-bound α-IgM for 24 hours. (**B**) Percentage of 2-NBDG internalized by CLL/N^{WT} (n = 5) and CLL/N^{M} (n = 7) (left) and MEC-1/N^{WT} (n = 10) and MEC-1/N^{M} cells (n = 13) (right) either unstimulated (-) or stimulated (+) with plate-bound α-IgM for 24 hours. (p ≤ 0.05, *; p ≤ 0.01, **; p ≤ 0.001, ***; p ≤ 0.0001, ****).

Supplementary Fig. 10. *NOTCH1*-Mediated Transcriptional Regulation of *TFAM*

(**A**) Genome browser tracks (visualized in IGV) showing CUT&Tag data for the NICD domain of NOTCH1 at the *TFAM* locus in wild-type (MEC-1/N^{WT}) and *NOTCH1*-mutant (MEC-1/N^{M})

CLL cells. Each track represents the merged signal from three biological replicates (n = 3) of aIgM treated MEC-1/N^{WT} and MEC-1/N^{M} samples. Histone modifications (H3K4me1, H3K4me3, H3K27ac, H3K27me3) and IgG (as control) are also shown to provide chromatin context. The lower panels display peaks called with SEACR for each histone mark and NICD, highlighting regions of significant enrichment. Numbers indicate the value of intensity of the peaks. (**B**) RNA-seq analysis of *NOTCH1*-mutant (MEC-1/N^{M}) and wild-type (MEC-1/N^{WT}) CLL cells under untreated (NT) and α-IgM–stimulated conditions. Bar plot displays trimmed mean of M-values (TMM-normalized counts) for the TFAM gene, highlighting differences in expression across conditions and genotypes. (**C**) *TFAM* silencing efficiency expressed as percentage of residual TFAM protein signal relative to siCTRL condition (100%) in MEC-1/N^{WT} and MEC-1/N^{M} cells (n = 3). (**D**) Representative confocal microscopy images of MEC-1/N^{WT} and MEC-1/N^{M} cells transfected with siCTRL or siTFAM and stained with Hoechst (blue) and MitoTracker Red (red) to visualize nuclei and mitochondria, respectively. (**E**) (left) Representative 3D reconstructions of mitochondria in siCTRL and siTFAM MEC-1/N^{WT} and MEC-1/N^{M} cells, generated using Imaris software on confocal Z-stacks, (right) quantification of mitochondrial surface volume from multiple independent images of MEC-1/N^{WT} and MEC-1/N^{M} cells following *TFAM* knockdown.

Supplementary Fig.11. Glutamine blockade enhances Venetoclax sensitivity in *NOTCH1*-mutated cells

(**A**) Heatmap showing the results of the combination index between V-9302 and venetoclax. (**B**) Percentage of viable MEC-1/N^{WT} and MEC-1/N^{M} cells assessed by Annexin V/7-AAD staining in standard conditions or, after the following conditions: untreated (UN), V-9302, UK-5099, venetoclax, V-9302 + venetoclax, and UK-5099 + venetoclax, for 72h (**C**) Percentage of CLL/N^{WT} and CLL/N^{M} cells in standard conditions or, after the following

conditions : untreated (UN), V-9302, venetoclax and V-9302 + venetoclax for 24h (CLL/N^{WT} n = 5, CLL/N^{M} n = 8). ($p \leq 0.05$, *; $p \leq 0.01$, **; $p \leq 0.001$, ***; $p \leq 0.0001$, ****).

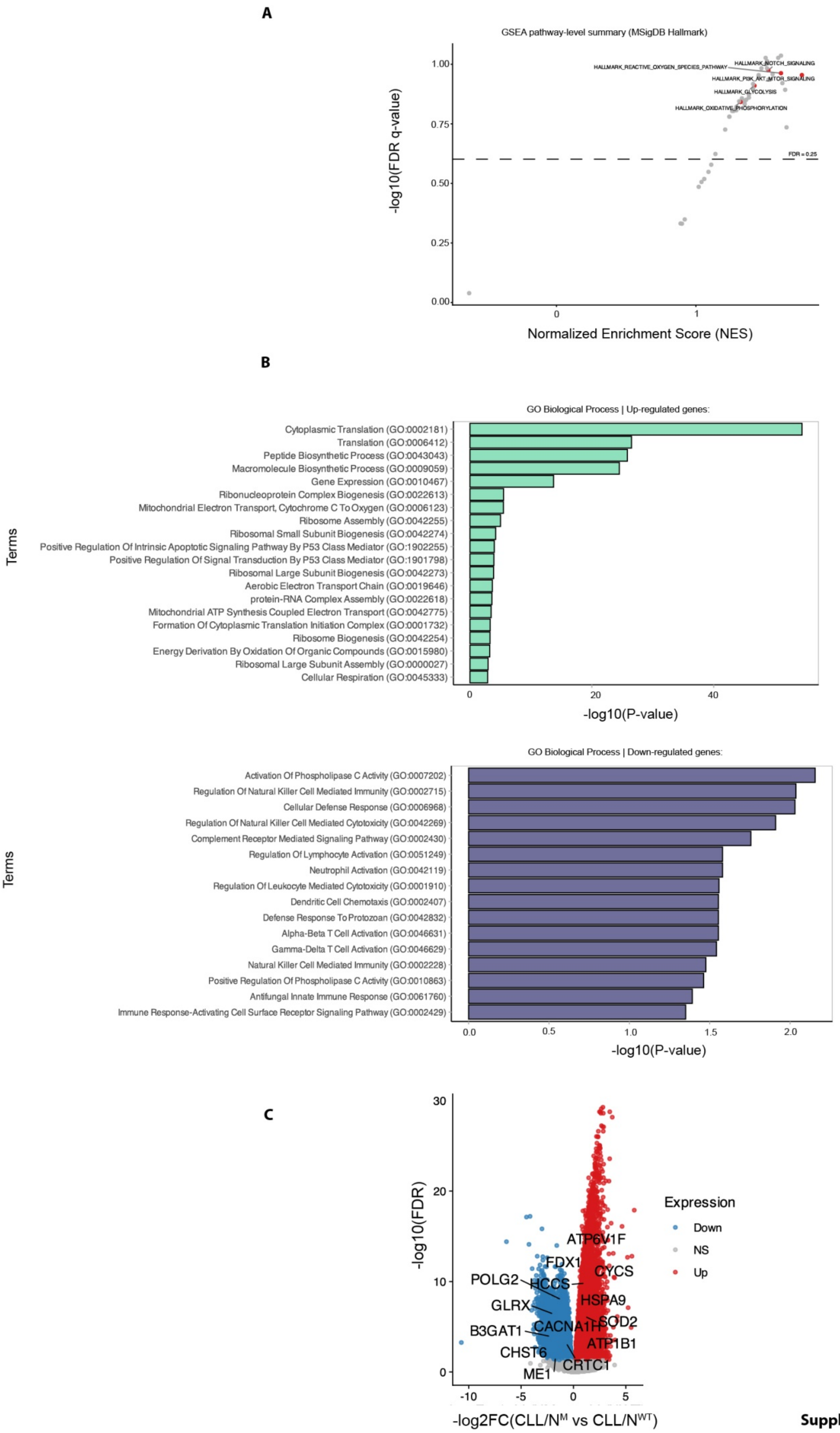

Supplementary Fig.1

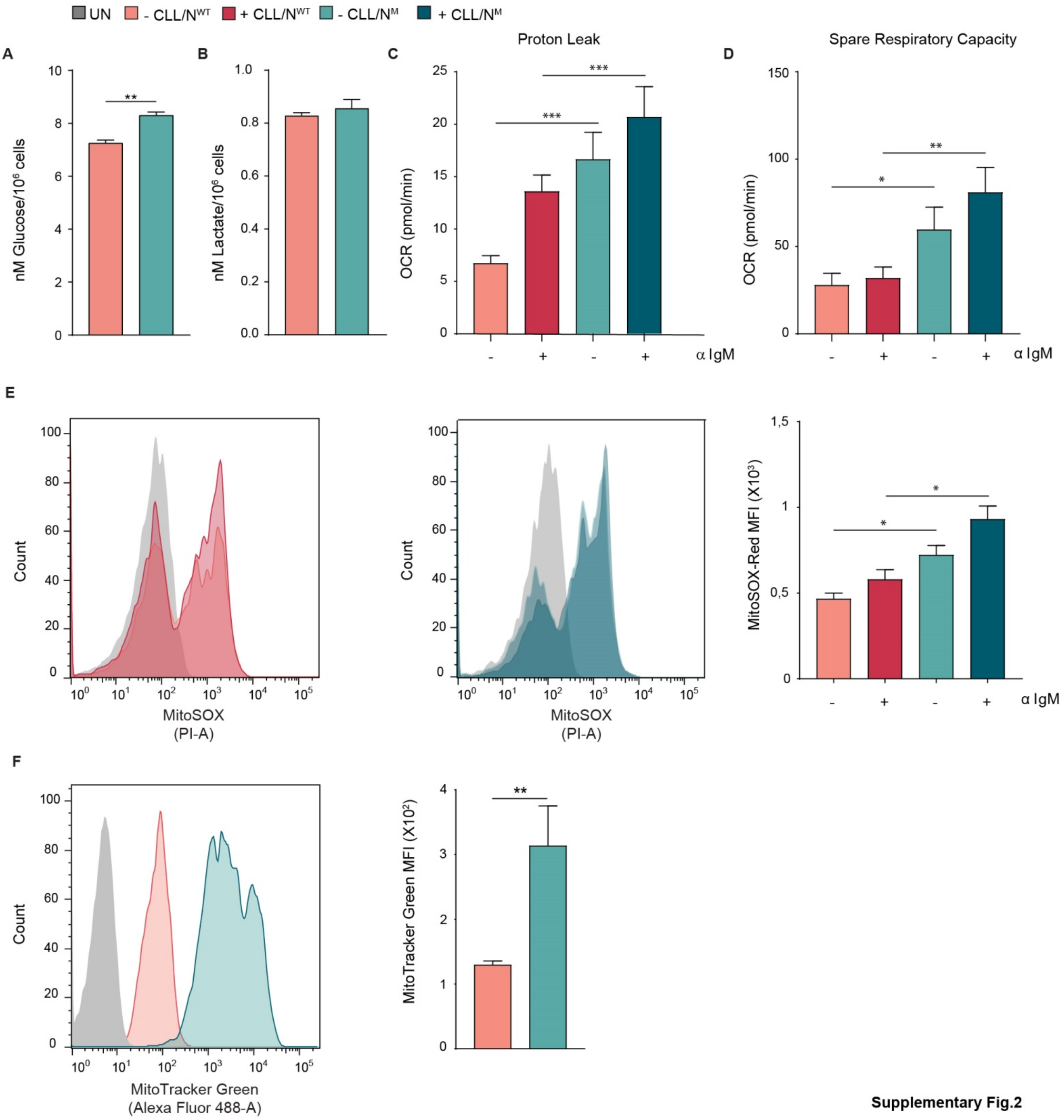

544

Supplementary Fig.2

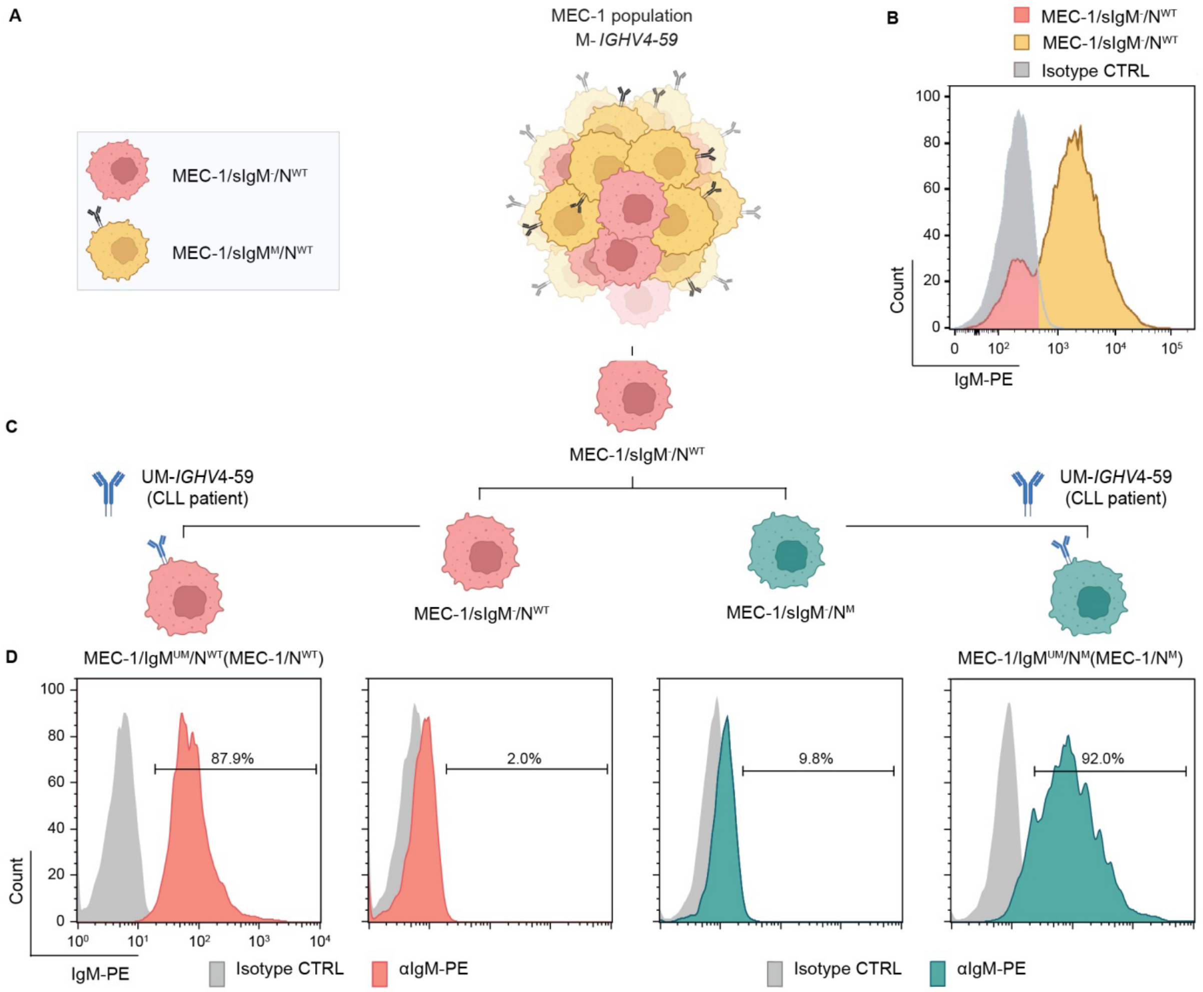

545 Supplementary Fig.3

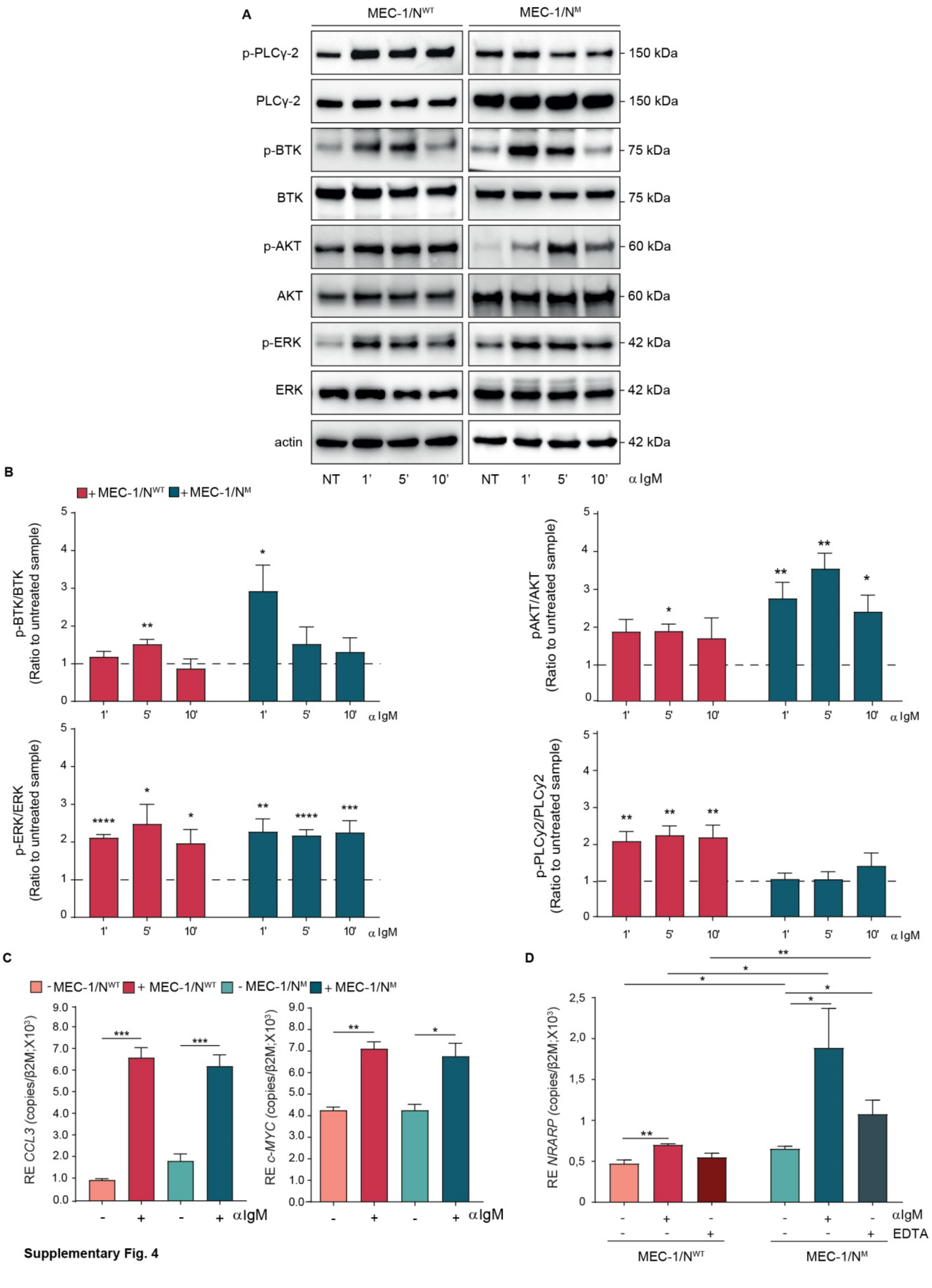

546

Supplementary Fig. 4

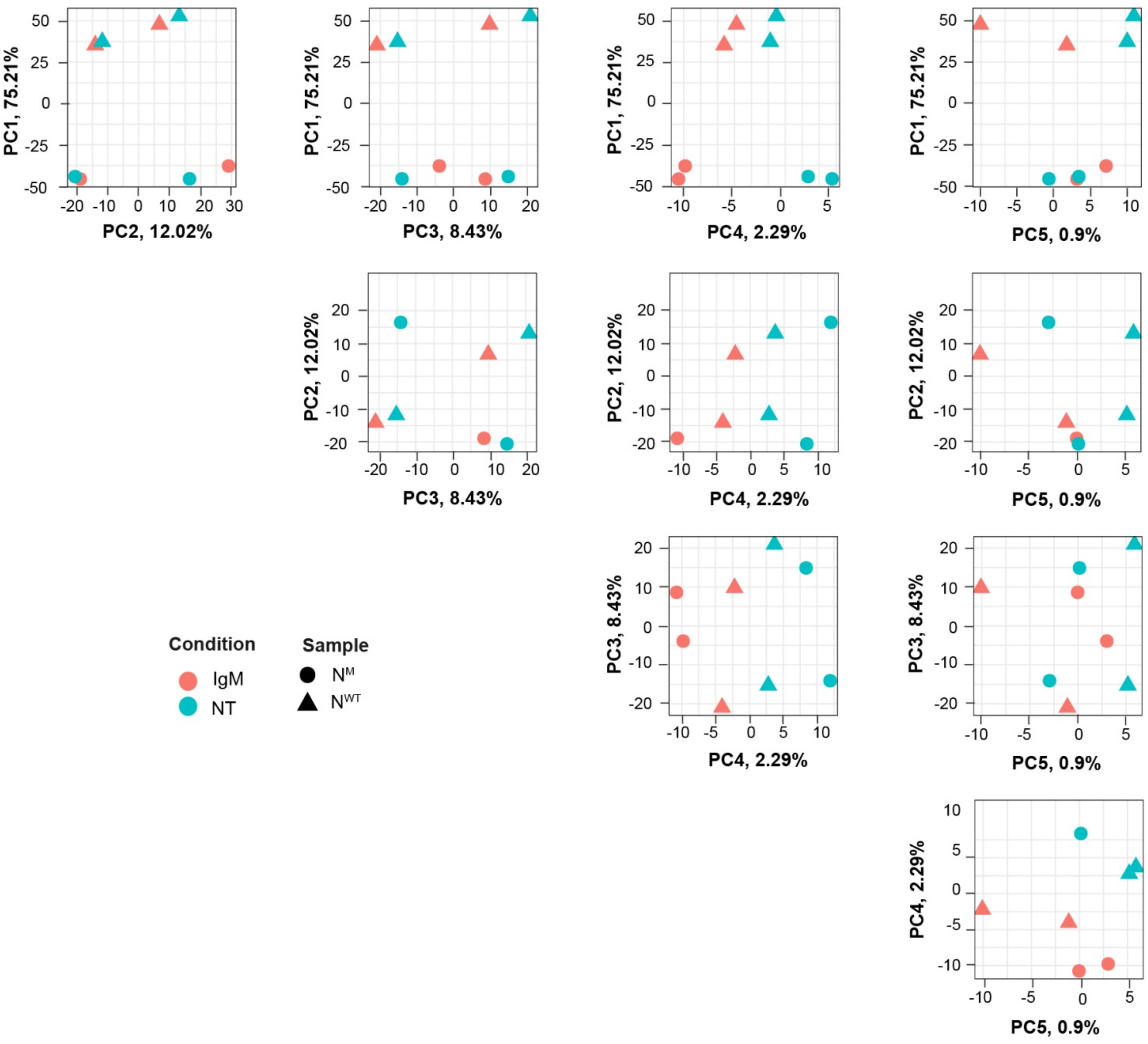

Supplementary Fig. 5

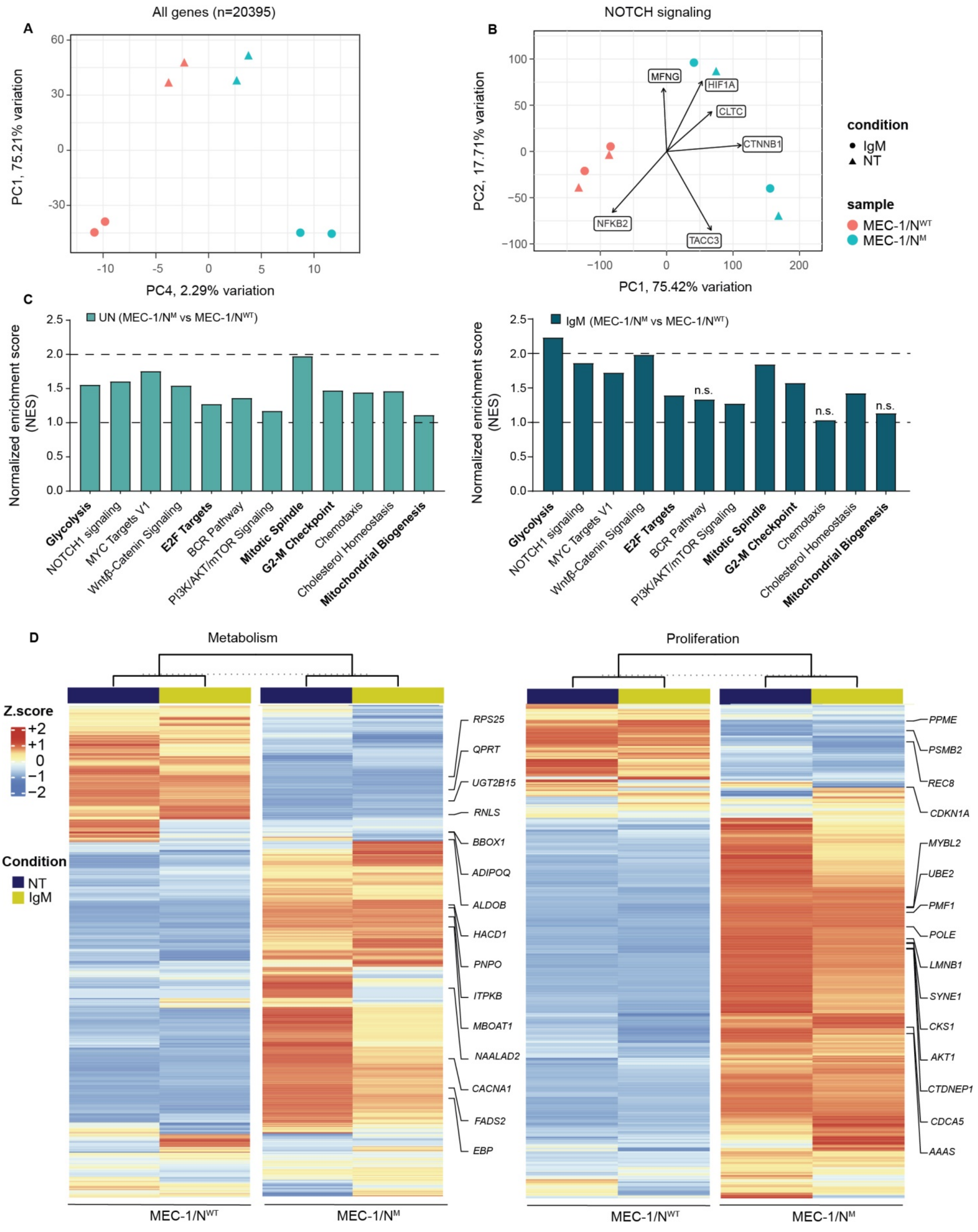

548

Supplementary Fig.6

MEC-1/N[M] vs MEC-1/N[WT]

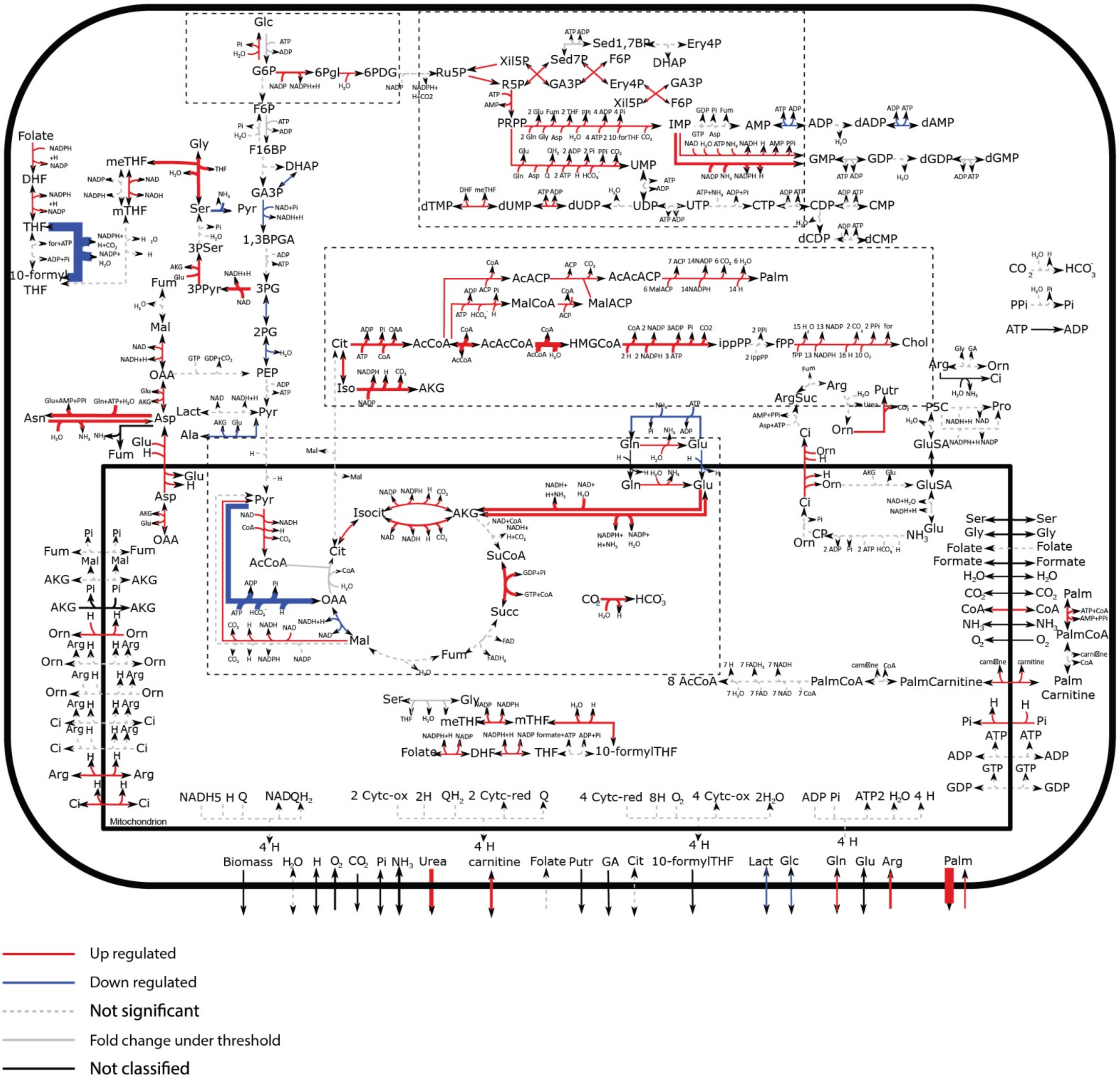

Supplementary Fig.7

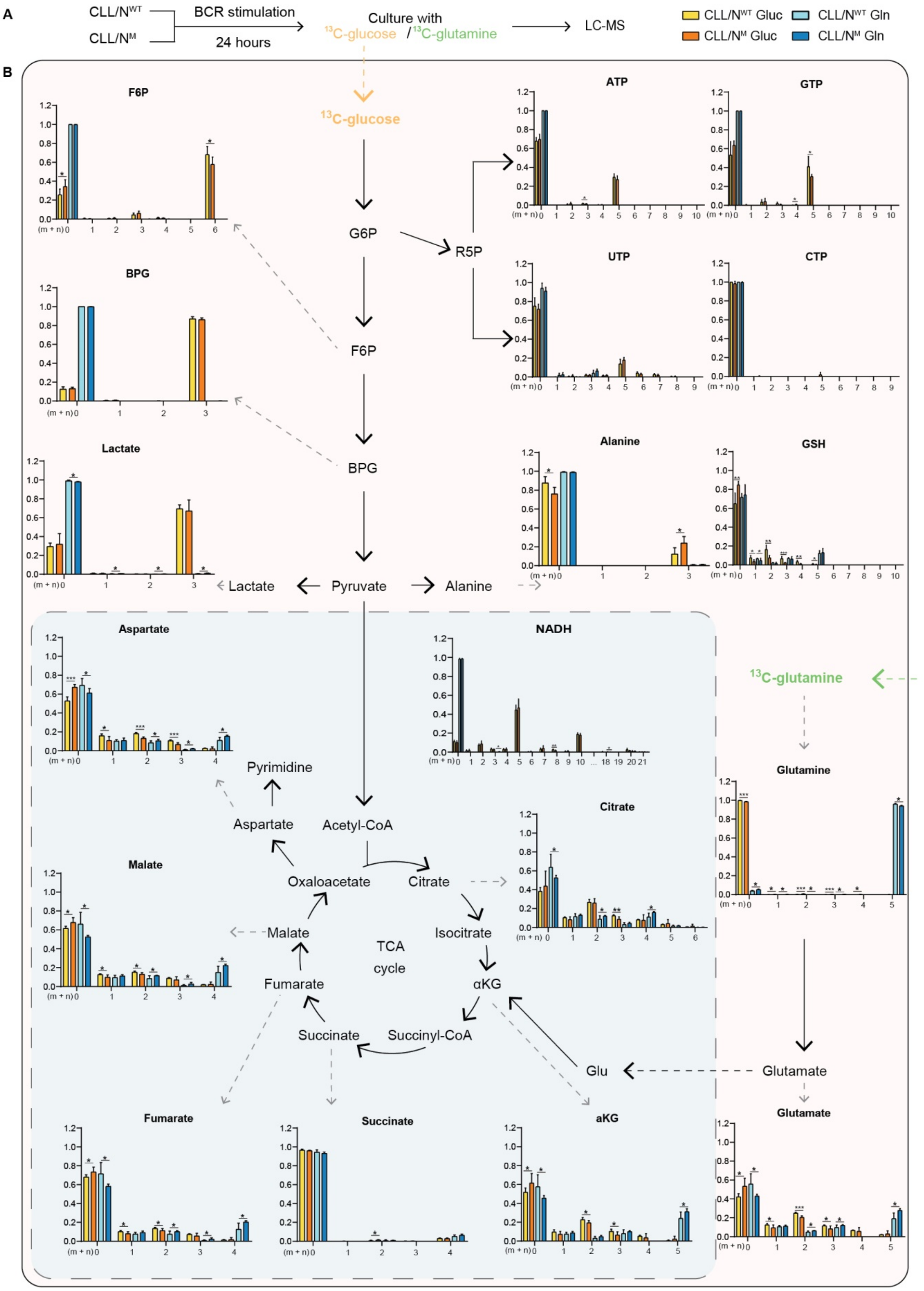

Supplementary Fig.8

550

A

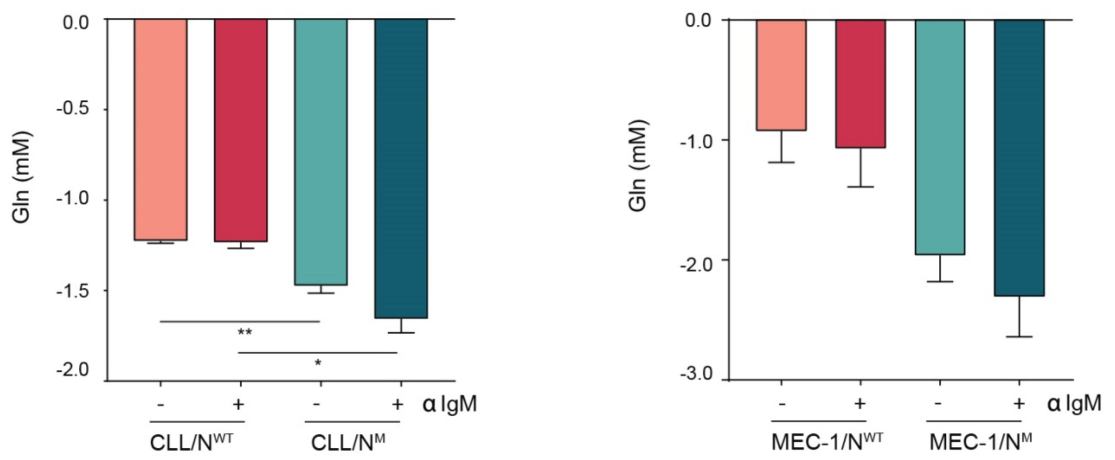

B

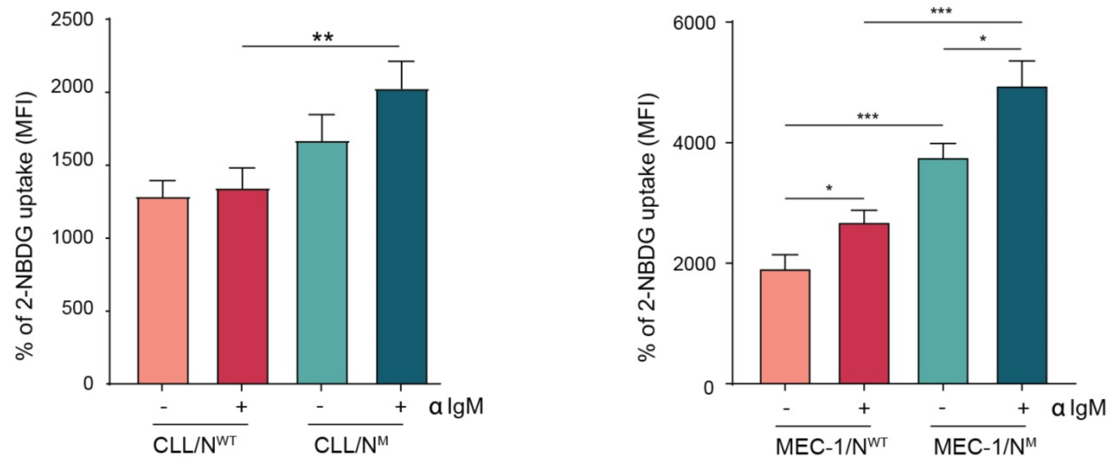

551

Supplementary Fig.9

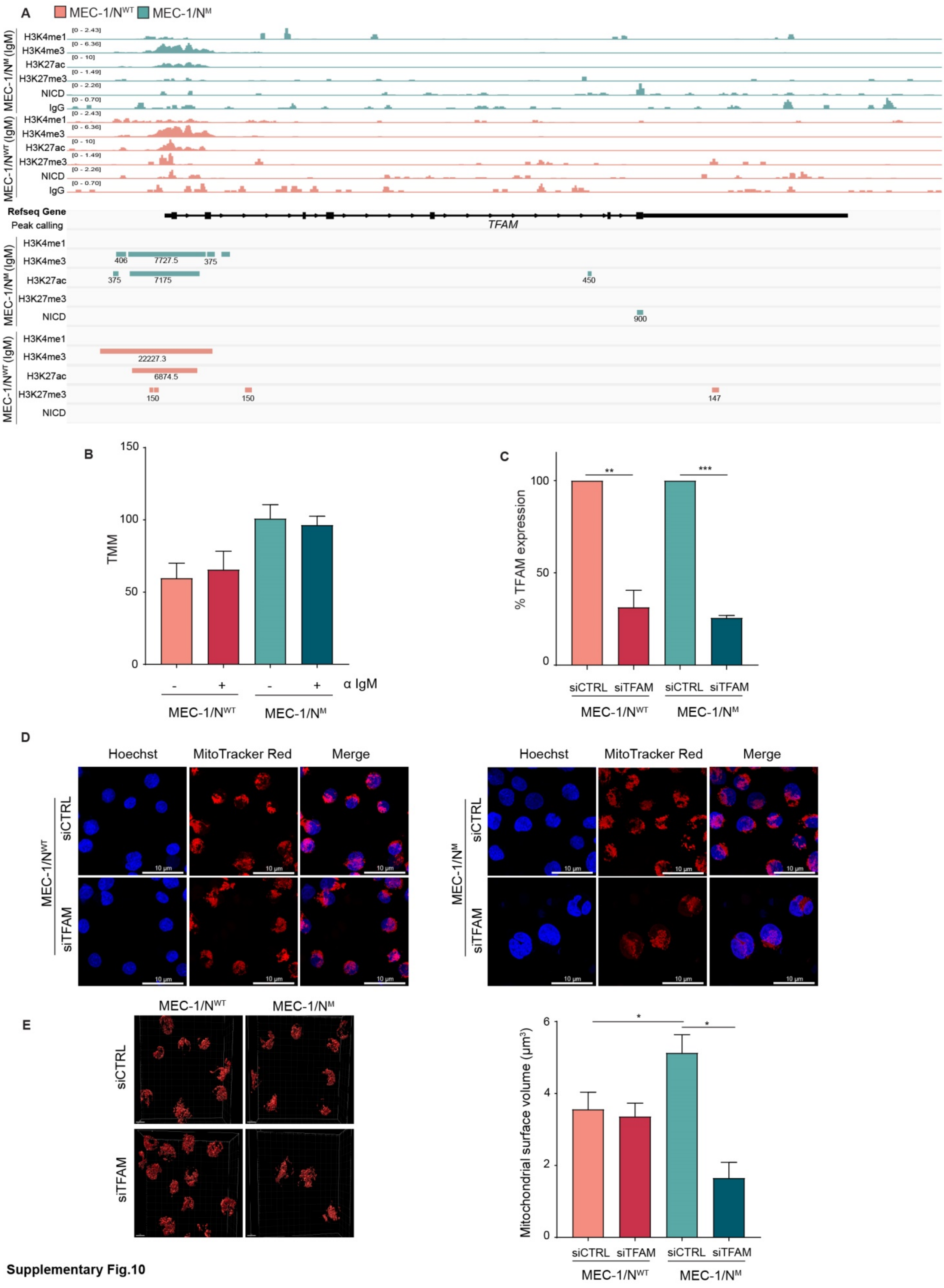

Supplementary Fig.10

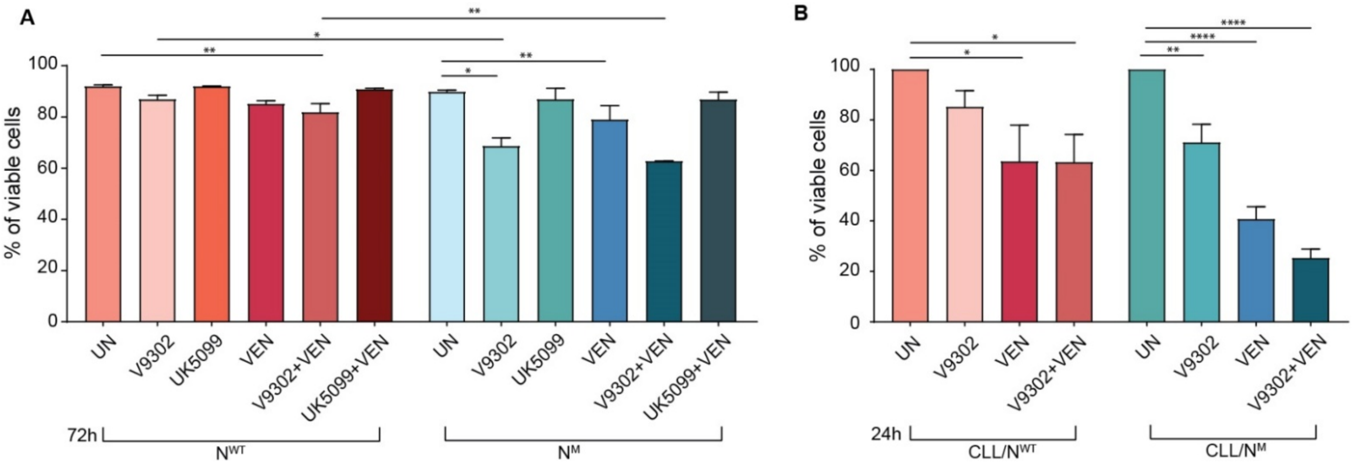

553

Supplementary Fig.11
